# Impact of Backbone Substitution on Organocatalytic Activity of Sterically Encumbered NHC in Benzoin Condensation

**DOI:** 10.3390/molecules29081704

**Published:** 2024-04-10

**Authors:** Rinat R. Aysin, Konstantin I. Galkin

**Affiliations:** 1 A. N. Nesmeyanov Institute of Organoelement Compounds, Russian Academy of Sciences, Vavilova Street 28, bld. 1, 119991 Moscow, Russia; aysin@ineos.ac.ru; 2 N. D. Zelinsky Institute of Organic Chemistry, Leninsky Prospect 47, Russian Academy of Sciences, 119991 Moscow, Russia; 3 Laboratory of Green Chemistry, Bauman Moscow State Technical University, 2nd Baumanskaya Street 5/1, 105005 Moscow, Russia

**Keywords:** benzoin condensation, organocatalysis, *N*-heterocyclic carbenes, furfural, density functional calculations

## Abstract

In this study, we provide a theoretical explanation for the experimentally observed decrease in the organocatalytic activity of *N*-aryl imidazolylidenes methylated at the C4/5-H positions in the benzoin condensation of aromatic aldehydes. A comparative quantum chemical study of energy profiles for the NHC-mediated benzoin condensation of furfural has revealed a high energy barrier to the formation of the IPr^Me^-based furanic Breslow intermediate that can be attributed to the negative steric interactions between the imidazole backbone methyl groups and *N*-aryl substituents.

## 1. Introduction

**Scheme 1:**
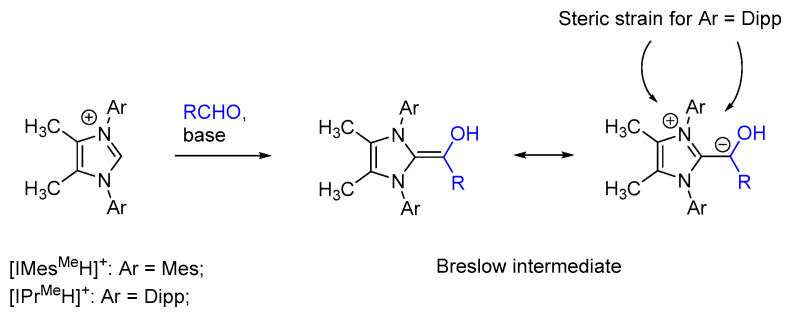
Carbonyl umpolung via the formation of the Breslow intermediate from C4,5-dimethylated *N*-aryl NHC. This work describes the steric impact of imidazole backbone substitution in NHC on organocatalytic activity in benzoin condensation. Mes = 2,4,6-trimethylphenyl, Dipp = 2,6-diisopropylphenyl.

*N*-heterocyclic carbenes (NHCs) have emerged as efficient organocatalysts, finding wide applications in various synthetic processes [1,2,3,4,5]. One such reaction of interest is the NHC-mediated benzoin condensation of aldehydes, which leads to valuable benzoin-type products that serve as building blocks for fine chemicals, monomers, and high-energy alkane biofuels [6,7,8]. The key intermediate in this carbonyl umpolung reaction is the Breslow intermediate (BI), which forms through a proton transfer in the NHC–aldehyde adduct by several distinct mechanisms [9,10,11,12]. The BI can be represented by two resonance forms: enaminol (the BI itself) and a zwitterionic moiety, which is an acyl carbanion equivalent (Scheme 1) [11,13]. Different types of NHCs, including “normal” azolium-based carbenes and “abnormal” 1,2,3-triazolylidenes, have been employed in benzoin condensation [8,14].

While imidazole-2-ylidenes are well known as organocatalysts [3,5], the catalytic activity of C4,5-substituted imidazole-2-ylidenes has been investigated less often. It may be expected that the substitution of the C4,5-H protons in imidazolium skeleton by electron-donor substituents enhances the electronic donor properties and nucleophilicity of the C2 carbene atom, thereby providing the increase in the organocatalytic activity of C4,5-substituted NHC [15,16,17,18]. Previously, it was demonstrated that C4,5-substituted NHCs can act as efficient organocatalysts for various reactions, such as nucleophilic aroylation of fluorobenzenes [19], stereoselective ring-opening polymerization of lactide [20], or conjugate umpolung of α,β-unsaturated aldehydes [21]. On the other hand, the decreased organocatalytic activity of some C4,5-substituted *N*-aryl imidazole-2-ylidenes have been revealed in benzoin condensation [22] and reaction of *trans*-cinnamaldehyde with *para-*chlorobenzaldehyde [15]. In our previous work, we showed the thermodynamic possibility of a non-classical mechanism of NHC-mediated benzoin condensation of aromatic aldehydes, which involves the generation of a C4-formed carbanionic carbene Breslow intermediate (CCBI) [23]. This mechanism is based on ambident reactivity of imidazole-2-ylidenes by both C2 and C4 carbene positions, and was found to be particularly attractive for reactions involving aromatic aldehydes and sterically encumbered *N*-aryl-substituted NHC [23,24,25].

To further investigate the mechanism of carbonyl umpolung and steric influence of imidazolium backbone substitution, we performed comparative experimental and theoretical studies on the benzoin condensation reaction with aromatic aldehydes mediated by 1,3-bis(2,4,6-trimethylphenyl)-imidazol-2-ylidene (IMes) and 1,3-bis(2,6-diisopropylphenyl)-imidazol-2-ylidene (IPr), and their C-4,5 dimethylated derivatives 1,3-bis(2,4,6-trimethylphenyl)-4,5-dimethyl-imidazol-2-ylidene (IMes^Me^) and 1,3-bis(2,6-diisopropylphenyl)-4,5-dimethyl-imidazol-2-ylidene (IPr^Me^) (Scheme 1). The DFT calculations on the mechanism of NHC-mediated benzoin condensation of furfural showed significant energy barriers to the formation of the Breslow intermediate from IPr^Me^ and its subsequent condensation with a second furfural molecule. These barriers were attributed to unfavorable steric interactions between the methyl groups on the imidazole backbone and the *N*-Dipp substituents.

## 2. Results and Discussion

To evaluate the role of imidazole backbone substitution, we conducted a comparative study on the catalytic activity of IMes, IPr and their C4,5-dimethylated derivatives IMes^Me^ and IPr^Me^ in the benzoin condensation reaction with aromatic aldehydes (Table 1). The NHCs were generated in situ in the H-bonded form by deprotonating the appropriate imidazolium cations [8,26]. The reactions were conducted either under ambient conditions for 24 h or at 80 °C for 3 h in deuterated solvents bubbled with argon. To prevent oxidation side processes during isolation, the conversions of aldehydes and yields of benzoin condensation products were assessed through ^1^H nuclear magnetic resonance (NMR) analysis of crude reaction mixtures (isolated yields were not quantified). Results of reactions with IMes and IPr were obtained from reference [23]. The best results were observed for the reactions of IMes and IMes^Me^ with furfural (entries 1, 3) or 5-methylfurfural (entries 7, 8) under ambient conditions with DBU as a base, resulting in >90% conversion and >50% yields of the corresponding C10 or C12 furoins. Catalytic activity of IPr in the reactions involving furfural and 5-methylfurfural was slightly lower (entries 4, 9). The most effective catalytic activity towards benzaldehyde and *m*-anisaldehyde was achieved with the least sterically hindered IMes carbene, giving corresponding benzoin-type products with 24% and 41% yields, respectively (entries 10, 13). The more sterically hindered IPr (entries 12, 15) and C4,5-dimethylated IMes^Me^ (entries 11, 14) carbenes displayed significantly lower catalytic activity towards benzaldehyde and *m*-anisaldehyde. The variance in catalytic activity was due to the highly hindered IPr^Me^, which was inactive in the benzoin condensation towards the aromatic aldehydes used under the employed conditions (entries 16–20). These experimental results suggest a significant decrease in the organocatalytic activity of highly sterically encumbered *N*-aryl NHC in benzoin condensation after backbone substitution.

**Scheme 2:**
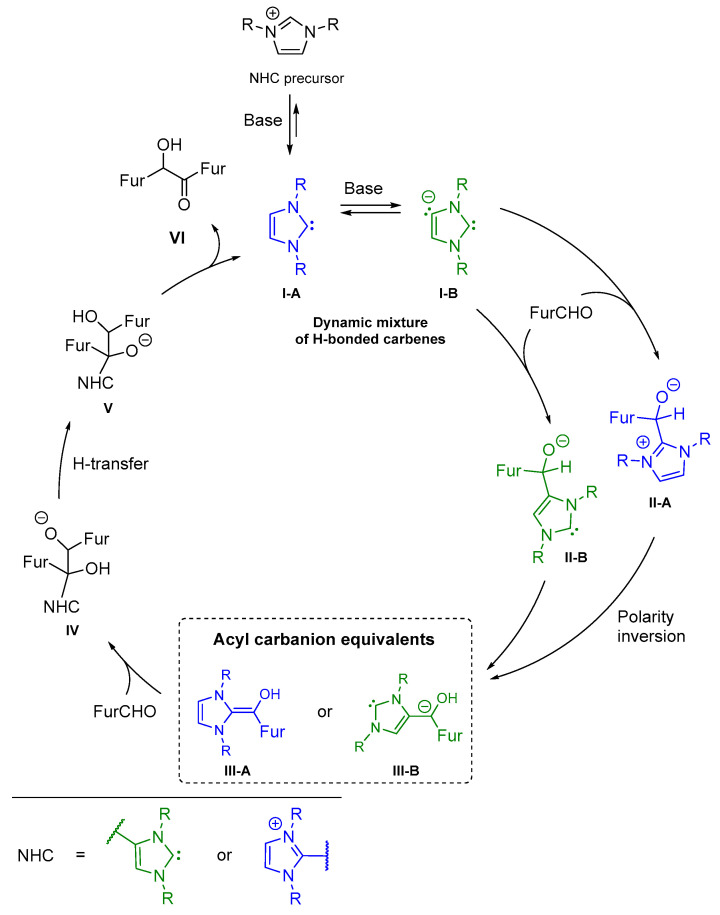
Adaptive dynamic mechanism of the benzoin condensation catalyzed by imidazolidinyl carbenes. The blue color indicates the C2 pathway, while the green color signifies the C4 pathway. Fur = 2-furyl. H-bonding is omitted for clarity. Adapted from [23] (with permission from the Royal Society of Chemistry, 2023).

According to our previously reported mechanistic evidence, the benzoin condensation of furfural mediated by C4,5-unsubstituted imidazolidinyl carbenes may proceed via an adaptive dynamic mechanism involving both C2 and C4 positions of the imidazolium ring (Scheme 2) [23]. Deprotonating the C2-H position in the azolium cation with a base results in the formation of a normal (C2) carbene, with or without a significant energy barrier, depending on the specific base employed [23,24]. The following activation of the C4-H bond in the imidazolium cation generates a dynamic mixture of the H-bonded NHC (I-A) and H-bonded ditopic carbanionic carbene (*dc*NHC, I-B). The nucleophilic attack of the carbonyl group of the aldehyde can be carried out at either the C2 or C4 position of the carbene. Depending on this direction, the subsequent polarity inversion involves the formation of adduct II-A, followed by base-mediated deprotonation of BI (III-A) or the formation of an anionic carbene II-B and then generation of CCBI (III-B). The next condensation stage leads to the formation of adduct IV, which subsequently rearranges into V. The following elimination stage results in the production of benzoin-type products VI, with the recovery of H-bonded carbene I(A-B).

**Figure 1:**
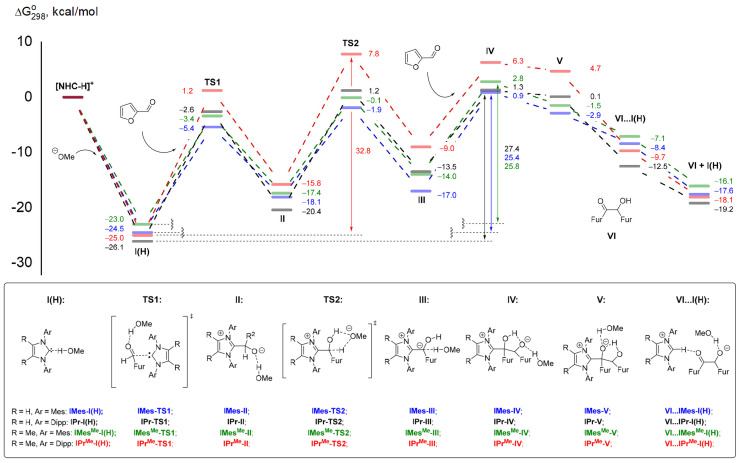
The DFT-calculated free energy profiles for the benzoin condensation of furfural mediated by IMes (blue), IPr (red), IMes^Me^ (green) and IPr^Me^ (gray). DFT calculations for pathways based on IMes and IPr were obtained from reference [23]. “‡” is the standard designation of the transition state.

Modification of the C4,5-H protons within the imidazolium core impacts the catalytic performance of NHC by altering the nucleophilicity of the C2 carbene atom and the stability of the Breslow intermediate [15,27]. It may be expected that substituting the C4,5-H protons in the imidazolium skeleton with electron-donor groups will enhance the electronic donation properties and nucleophilicity of the C2 carbene atom, consequently leading to an increase in the organocatalytic activity of C4,5-substituted NHCs [15]. The observed decrease in the catalytic activity of NHC after methylation at the C4/5 positions may be attributed to two possible reasons. Firstly, the negative steric effect between the imidazole backbone methyl groups and *N*-aryl substituents in NHC may hinder the formation of the catalytically active species, making it thermodynamically more challenging. On the other hand, the catalytic inactivity of IPr^Me^, along with the high catalytic activity of C4/5-unsubstituted IPr, may suggest that the benzoin condensation reaction catalyzed by very sterically demanding NHCs predominantly proceeds through an alternative pathway involving only the C4 position of the imidazolium core. To address these questions, we employed DFT calculations (details in ESI) to compare the energy profiles of the benzoin condensation reaction of furfural using H-bonded NHCs. Figure 1 illustrates the free energy profile and the chemical structures of the intermediates and transition states, starting from [IMes^Me^H]^+^ and [IPr^Me^H]^+^ with a methoxide anion as a base in comparison with energy profiles of [IMesH]^+^- and [IPrH]^+^-mediated reactions, which have been reported previously [23].

The free energy values and chemical structures of the intermediates and transition states for the benzoin condensation of furfural mediated by IMes, IPr, IMes^Me^, and IPr^Me^ are depicted in Figure 1. Based on DFT calculations, the adduct IMes-II and the Breslow intermediate IMes-III exhibit similar stability and activation energy barriers when compared to IMes^Me^-II and IMes^Me^-III, respectively, with differences in energy values around ±3 kcal mol^−1^. The nucleophilic attack of III to a second molecule of furfural represents the limiting stage for both IMes and IMes^Me^ pathways, characterized by activation energy barriers ΔG^≠^_IMes-I(H)→IMes-TS2_ of 25.4 kcal mol^−1^ and ΔG^≠^_IMesMe-I(H)→IMesMe-TS2_ of 25.8 kcal mol^−1^. The subsequent stages of furoin VI formation proceed with similar energy parameters for IMes- and IMes^Me^-derived intermediates V and VI…I(H). These results are consistent with the experimental data, suggesting similar organocatalytic activity of IMes and IMes^Me^ in the benzoin condensation of furfural.

In contrast, a comparison of the energy profiles for the benzoin condensation of furfural mediated by the sterically demanding IPr and IPr^Me^ reveals a significant difference in energy parameters for the two pathways. The addition of IPr^Me^-I(H) at the C2 carbene position to furfural has a 2.7 kcal mol^−1^ higher energy barrier compared to the addition of IPr-I(H). Furthermore, the resulting adduct IPr-II is 4.6 kcal mol^−1^ more stable than the adduct IPr^Me^-II. In both IPr and IPr^Me^ pathways, the base-mediated generation of the acyl carbanionic intermediates III is the limiting stage. The formation of IPr^Me^-III is characterized by a high energy barrier ΔG^≠^_IPrMe-I(H)→IPrMe-TS2_ of 32.8 kcal mol^−1^, which is 5.5 kcal mol^−1^ higher than ΔG^≠^_IPr-I(H)→IPr-TS2_. The resulting IPr-III and IPr-IV intermediates are 4.5 kcal mol^−1^ and 5.0 kcal mol^−1^ more stable than the IPr^Me^-III and IPr^Me^-IV intermediates, respectively.

The lower thermodynamic stability of the Breslow intermediate IPr^Me^-III in comparison to IPr-III, as well as a high activation energy barrier for its generation, can be explained by the undesirable steric effects of the imidazole backbone methyl groups. This steric influence decreases the availability of the C2 carbene position in IPr^Me^-I(H) and the “aldehydic” CH-position in IPr^Me^-III. Moreover, the steric influence of the additional methyl groups may be restricting rotation in IPr^Me^-III, thus having a downstream impact on its nucleophilicity.

## 3. Materials and Methods

### 3.1. General Procedure for Benzoin Condensation

A mixture of aldehyde (0.5 mmol), imidazolium chloride (0.05 mmol) and a base (0.5 mmol) in deuterated acetonitrile was treated at the appropriate temperature and time conditions (24 h at 22 °C or 3 h at 80 °C) under an argon atmosphere. The degree of conversion and the yield of benzoin condensation products were measured by NMR using PhSiMe_3_ as internal standard. The results of the reactions are summarized in Table 1.

### 3.2. Computational Details

The reaction energy profiles of the benzoin condensation of furfural (Scheme S1) were calculated using ORCA v.5 software [28,29] at the PBE0/Def2-TZVP level [30,31] in the frame of the CPCM solvation model [32,33,34,35] with MeOH parameters. For acceleration, the RIJCOSX approximation [36,37,38] with the Def2/j fitting [39] basis set was used. Frequencies and eigenvectors of normal modes were calculated in harmonic approximation for all the reaction participants. Virtual modes were absent for the intermediates, whereas one virtual mode was found for the transition states. The obtained reaction path repeats that previously proposed [23]. The calculated arbitrary energies for the reaction participants and optimized xyz Cartesian coordinates are presented in the Supporting Information (Section 3.2, Tables S1 and S2). The methylate base was chosen to simplify the calculation. The barrierless nature of the first stage and the addition of the second furfural to the intermediate III was proved by relaxed scan along with the C_2_–H distance (Figures S5 and S6). A very small barrier was found for the detachment of benzoin from the intermediate V and proved by PES scan along with the corresponding C…FurC(=O) distance (Figure S7). The estimated activation energies are very small (1 kcal/mol); therefore, this stage can be considered barrier-free, as indicated in Scheme S1.

## 4. Conclusions

The impact of imidazole backbone substitution on the organocatalytic activity of common *N*-aryl imidazolylidenes in the benzoin condensation of aromatic aldehydes was studied. Experiments consistent with the introduction of methyl groups on the NHC backbone led to a decrease in organocatalytic activity of IMes^Me^ and IPr^Me^ compared to their non-methylated counterparts IMes and IPr. Differences in reactivity were observed in reactions with IMes-derived carbenes and benzaldehydes, as well as with both furaldehydes and benzaldehydes when employing the more sterically demanding IPr-based carbenes. The DFT calculation results for the mechanism of the NHC-mediated benzoin condensation of furfural are in line with these experimental results and revealed substantial energy barriers to the formation of the Breslow intermediate derived from IPr^Me^ and the subsequent condensation with the second molecule of furfural. These barriers can be attributed to adverse steric interactions between the methyl groups on the imidazole backbone and the *N*-Dipp substituents.

## Figures and Tables

**Table 1:** A comparison of catalytic activity of IMes, IPr and C4/5-methylated IMes^Me^ and IPr^Me^ in benzoin condensation of aromatic aldehydes ^1^.

№	Imidazolium Salt, Base	Aldehyde	T (°C)	Conversion/Yield of Benzoin (%)
1	[IMesH]Cl, DBU	Furfural	22 °C	98/51
2	[IMesH]Cl, *^t^*BuONa	Furfural	22 °C	99/32
3	[IMes^Me^H]Cl, DBU	Furfural	22 °C	99/42
4	[IPrH]Cl, DBU	Furfural	22 °C	99/40
5	[IPrH]Cl, DBU	Furfural	80 °C	99/38
6	[IPrH]Cl, *^t^*BuONa	Furfural	22 °C	99/34
7	[IMesH]Cl, DBU	5-Methylfurfural	22 °C	93/63
8	[IMes^Me^H]Cl, DBU	5-Methylfurfural	22 °C	93/67
9	[IPrH]Cl, DBU	5-Methylfurfural	22 °C	93/53
10	[IMesH]Cl, DBU	Benzaldehyde	80 °C	77/24
11	[IMes^Me^H]Cl, DBU	Benzaldehyde	80 °C	29/6
12	[IPrH]Cl, DBU	Benzaldehyde	80 °C	53/5
13	[IMesH]Cl, DBU	*m*-Anisaldehyde	80 °C	84/41
14	[IMes^Me^H]Cl, DBU	*m*-Anisaldehyde	80 °C	23/9
15	[IPrH]Cl, DBU	*m*-Anisaldehyde	80 °C	31/14
16	[IPr^Me^H]Cl, DBU	Furfural	80 °C	29/0
17	[IPr^Me^H]Cl, *^t^*BuONa	Furfural	80 °C	48/0
18	[IPr^Me^H]Cl, DBU	5-Methylfurfural	80 °C	2/0
19	[IPr^Me^H]Cl, DBU	Benzaldehyde	80 °C	9/0
20	[IPr^Me^H]Cl, DBU	*m*-Anisaldehyde	80 °C	16/0

^1^ Reaction conditions: aldehyde (0.5 mmol), catalyst (0.05 mmol), base (0.05 mmol), CD_3_CN (0.5 mL), 24 h at 22 °C or 3 h at 80 °C.

## Data Availability

The original contributions presented in the study are included in the article/Supplementary Material. Further inquiries can be directed to the corresponding author.

## Supplementary Materials

The following supporting information can be downloaded at https://www.mdpi.com/article/10.3390/molecules29081704/s1. Table S1: Influence of methylation at the C-4,5 positions on catalytic activity of [IMesH]^+^ and [IPrH]^+^ in benzoin condensation of aromatic aldehydes; Figure S1: ^1^H NMR spectra of the reaction mixture (conditions from entry 2, Table S1); Figure S2: ^1^H NMR spectra of the reaction mixture (conditions from entry 3, Table S1); Figure S3: ^1^H NMR spectra of the reaction mixture (conditions from entry 6, Table S1); Figure S4: ^1^H NMR spectra of the reaction mixture (conditions from entry 9, Table S1); Scheme S1: The studied mechanism of benzoin condensation of furfural; Table S2: Arbitrary energy values for the benzoin condensation of furfural mediated by [IMes^Me^H]^+^- and [IPr^Me^H]^+^-derived catalytically active intermediates; Figure S5: PES scan for the [IMes^Me^H]^+^ deprotonation; Figure S6: PES scan for the IMes^Me^-III to IMes^Me^-IV stage; Figure S7: PES scan for the IMes^Me^-V to VI...IMes-I(H) stage.
