# Supplementary material for: Impact of Backbone Substitution on Organocatalytic Activity of Sterically Encumbered NHC in Benzoin Condensation
Source: Molecules. 2024 Apr 10;29(8):1704. doi: 10.3390/molecules29081704 (PMC11051995; doi:10.3390/molecules29081704)
Supplement: Supplementary file 1 [file molecules-29-01704-s001.zip › molecules-2917872-supplementary.pdf]

Supporting Information

for the article

The Impact of the Backbone Substitution on the Organocatalytic Activity of Sterically Encumbered NHC in Benzoin Condensation

Rinat R. Aysin and Konstantin I. Galkin

Table of Contents

1. General Information........ 2
2. Mechanistic studies........ 2
   2.1. Experimental study on benzoin condensation........ 2
   2.2. Selected NMR spectra of reaction mixtures........ 4
3. DFT calculations........ 6
   3.1. Computational details........ 6
   3.2. Optimized xyz-Cartesian coordinates........ 9

1. General Information

Commercially available starting compounds, reagents, and solvents were of analytical grade upon purchase or purified prior to use by standard methods. Imidazolium salts [IMes^{Me}H]Cl and [Pr^{Me}H]Cl were obtained by using published synthetic protocols (*New J. Chem.* **2017**, *41*, 1057-1063). NMR spectra were recorded using a Bruker Avance 300 spectrometer operating at 300.1 MHz for ^{1}H and 75.5 MHz for ^{13}C. NMR chemical shifts were measured relative to residual solvent peaks. The processing was carried out using the MestReNova software.

2. Mechanistic studies

2.1. Experimental study on benzoin condensation

General procedure for benzoin condensation.

A mixture of aldehyde (0.5 mmol), imidazolium chloride (0.05 mmol) and a base (0.5 mmol) in deuterated acetonitrile was treated at the appropriate temperature and time conditions (24 h at 22 °C or 3 h at 80 °C) under an argon atmosphere. The degree of conversion and the yield of benzoin condensation products were measured by NMR using $PhSiMe_3$ as internal standard. The results of the reactions are summarized in Table S1.

Table S1. Influence of methylation at the C-4,5 positions on catalytic activity of [IMesH]$^+$ and [IPrH]$^+$ in benzoin condensation of aromatic aldehydes.

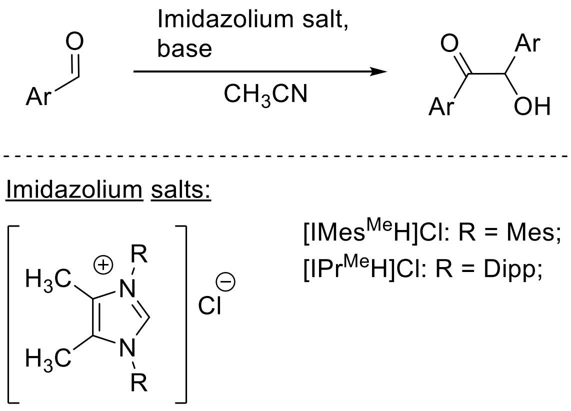

№	Imidazolium salt, base	Aldehyde	T (°C)	Conversion/yield of benzoin (%)[a]
1	[IMes^{Me}H]Cl, DBU	Furfural	22 °C	99/42
2	[IMes^{Me}H]Cl, DBU	5-Methylfurfural	22 °C	93/67
3	[IMes^{Me}H]Cl, DBU	Benzaldehyde	80 °C	29/6
4	[IMes^{Me}H]Cl, DBU	*m*-Anisaldehyde	80 °C	23/9
5	[IPr^{Me}H]Cl, DBU	Furfural	80 °C	29/0
6	[IPr^{Me}H]Cl, ^{t}BuONa	Furfural	80 °C	48/0
7	[IPr^{Me}H]Cl, DBU	5-Methylfurfural	80 °C	2/0
8	[IPr^{Me}H]Cl, DBU	Benzaldehyde	80 °C	9/0
9	[IPr^{Me}H]Cl, DBU	*m*-Anisaldehyde	80 °C	16/0

[a] Yields and conversions were determined by ^{1}H nuclear magnetic resonance (NMR) analysis of crude reaction mixtures (isolated yields were not determined). Consumption of two benzaldehyde molecules to yield one molecule of benzoin product has been accounted.

2.2. Selected NMR spectra of reaction mixtures

Figure S1. ^{1}H NMR spectra of the reaction mixture (conditions from entry 2, Table S1).

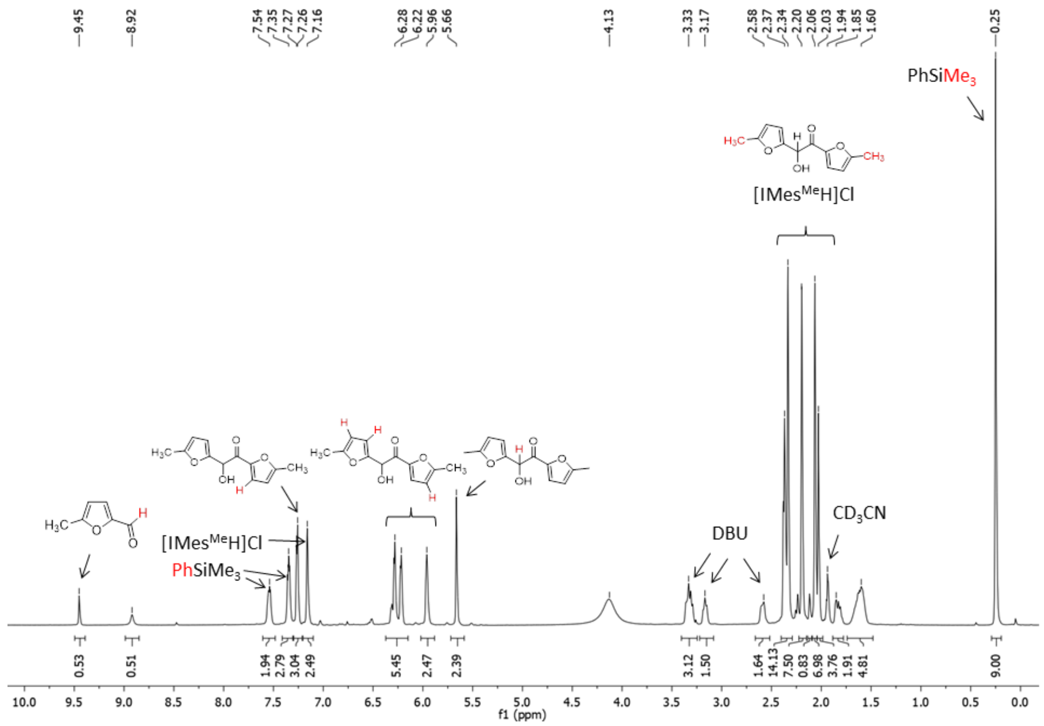

Figure S2. ^{1}H NMR spectra of the reaction mixture (conditions from entry 3, Table S1).

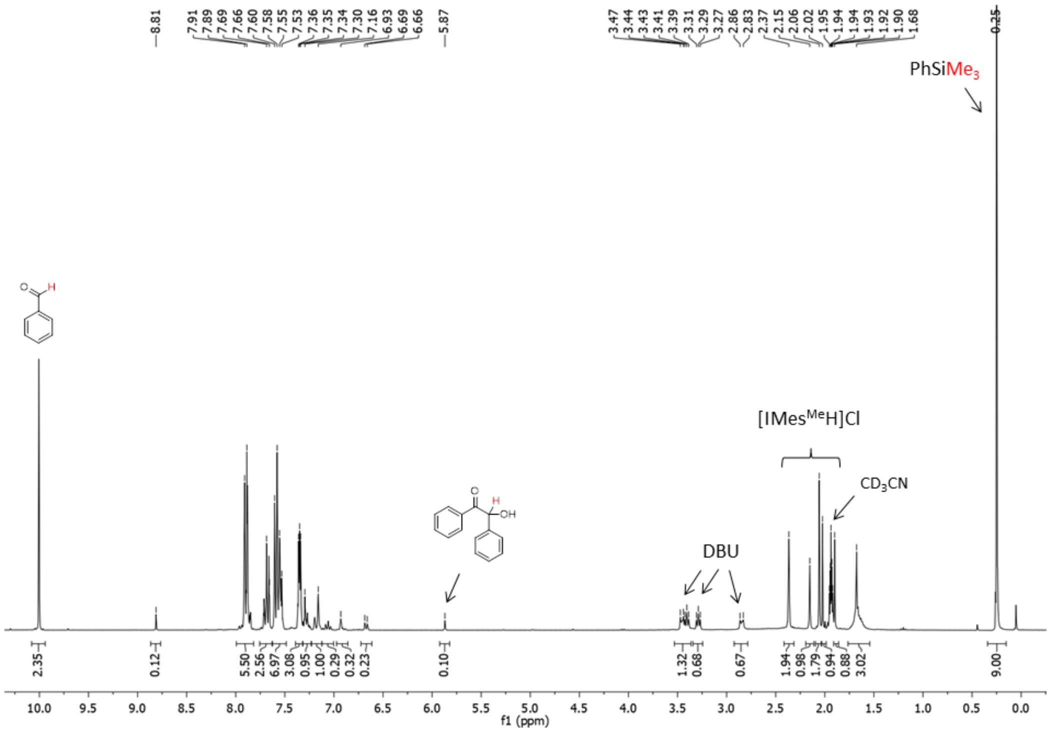

Figure S3. ^{1}H NMR spectra of the reaction mixture (conditions from entry 6, Table S1).

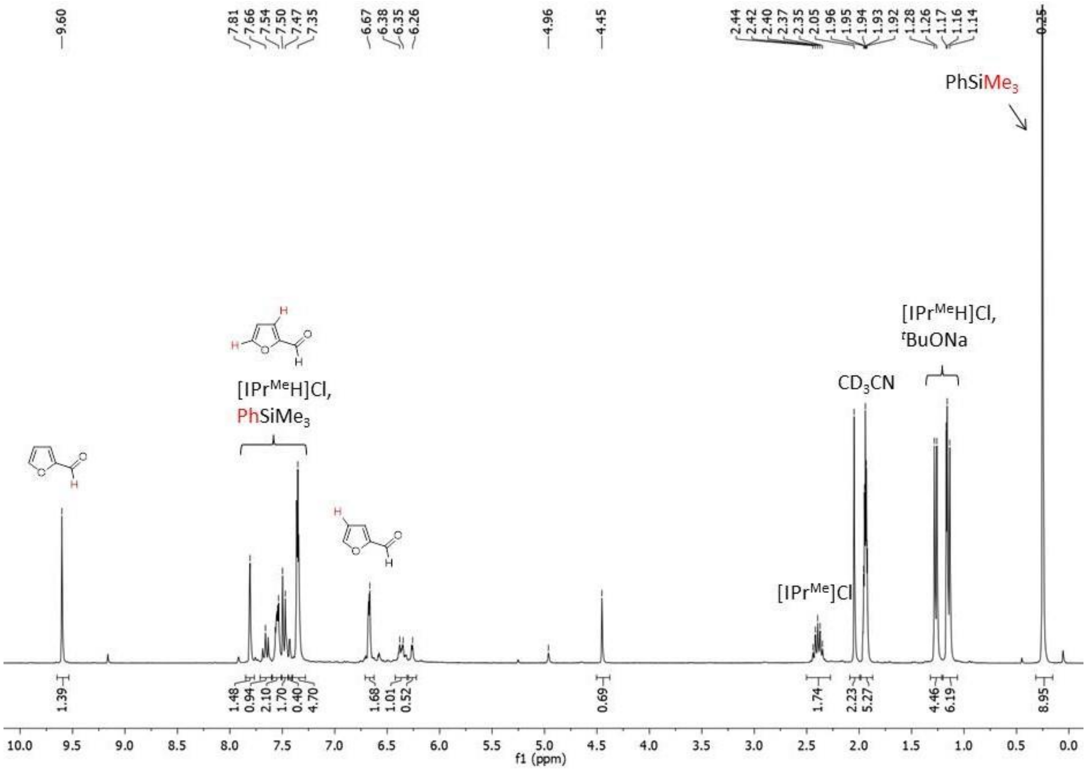

Figure S4. ^{1}H NMR spectra of the reaction mixture (conditions from entry 9, Table S1).

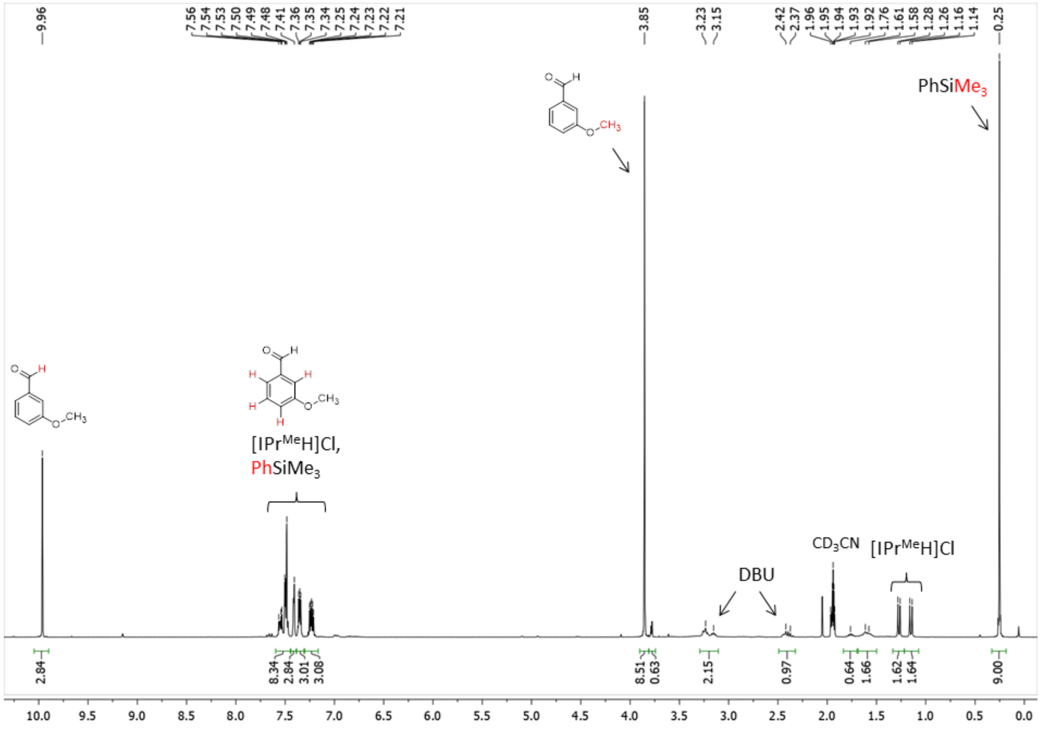

3. DFT calculations

3.1. Computational details

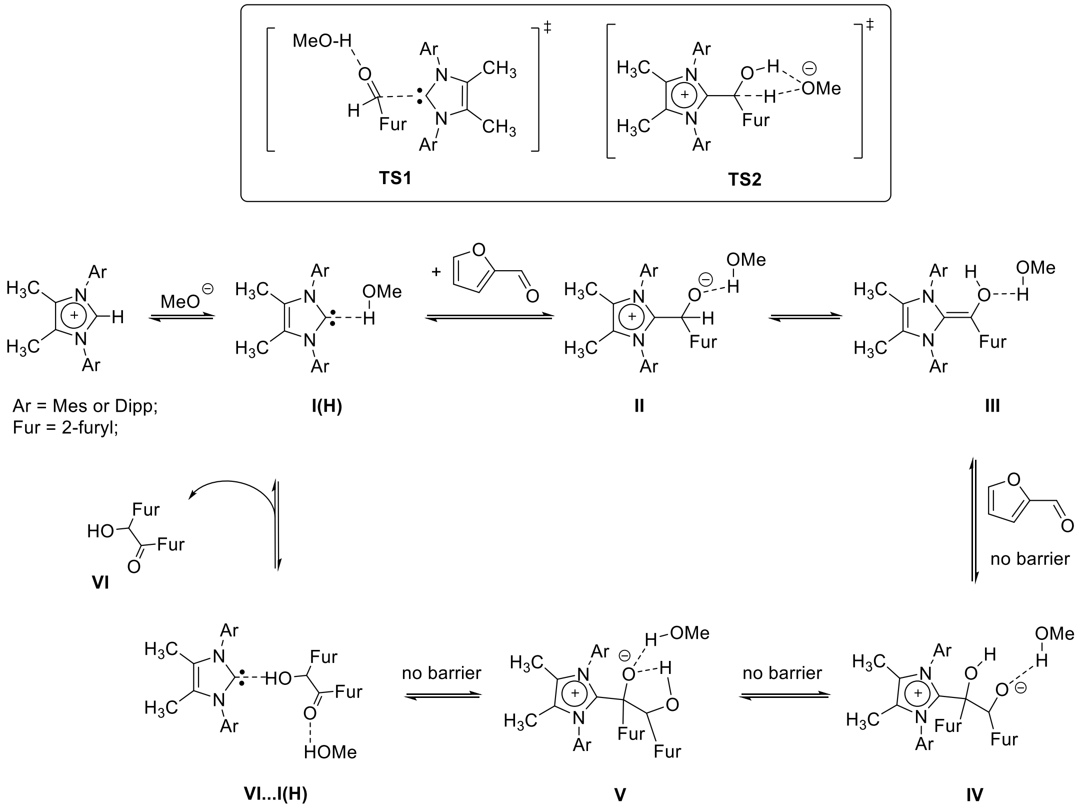

Scheme S1. The studied mechanism of benzoin condensation of furfural.

Table S2. Arbitrary energy values for the benzoin condensation of furfural mediated by $[IMes^{Me}H]^{+}$- and $[IPr^{Me}H]^{+}$-derived catalytically active intermediates.

Reaction participant	ΔE_{tot}, kcal/mol	ΔH^{o}_{298}, kcal/mol	ΔG^{o}_{298}, kcal/mol
**$[IMes^{Me}H]^{+}$**	0.0	0.0	0.0
**$IMes^{Me}$-I(H)**	-17.6	-16.8	-23.0
**$IMes^{Me}$-TS1**	-11.8	-10.7	-3.4
**$IMes^{Me}$-II**	-28.1	-28.4	-17.4
**$IMes^{Me}$-TS2**	-9.4	-8.5	-0.1
**$IMes^{Me}$-III**	-21.2	-18.9	-14.0
**$IMes^{Me}$-IV**	-23.5	-24.5	2.8
**$IMes^{Me}$-V**	-26.4	-26.4	-1.5
**VI...$IMes^{Me}$-I(H)**	-29.6	-28.8	-7.1
**VI + $IMes^{Me}$-I(H)**	-24.6	-24.7	-16.1

**[IPr^{Me}H]$^+$**	0.0	0.0	0.0
**IPr^{Me}-I(H)**	-19.2	-18.1	-25.0
**IPr^{Me}-TS1**	-7.2	-6.1	1.2
**IPr^{Me}-II**	-25.1	-24.5	-15.8
**IPr^{Me}-TS2**	-2.9	-3.1	7.8
**IPr^{Me}-III**	-18.2	-17.1	-9.0
**IPr^{Me}-IV**	-18.5	-18.5	6.3
**IPr^{Me}-V**	-19.9	-19.7	4.7
**VI... IPr^{Me}-I(H)**	-31.5	-30.0	-9.7
**VI + IPr^{Me}-I(H)**	-26.2	-26.0	-18.1

Figure S5. PES scan for the [IMes^{Me}H]$^+$ deprotonation.

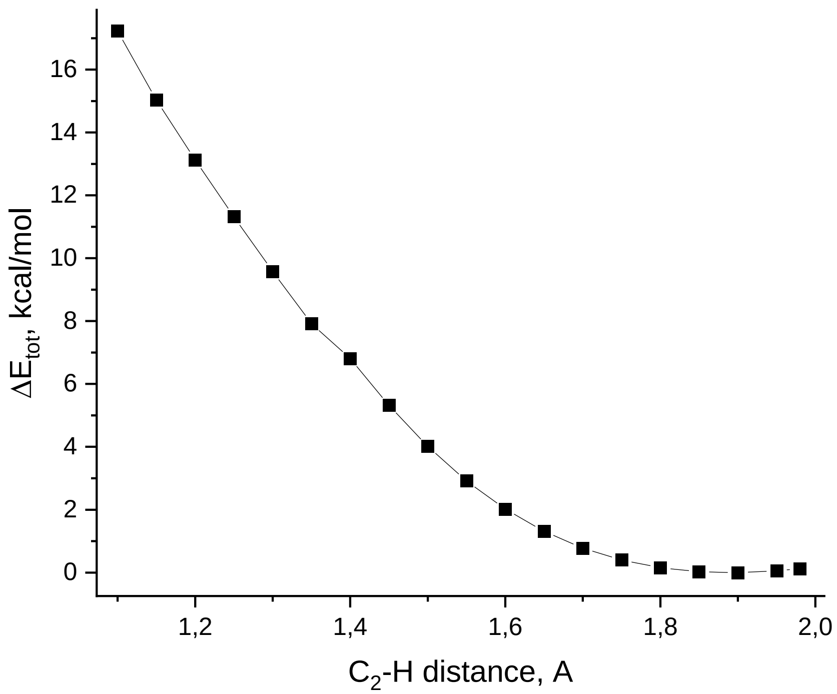

Figure S6. PES scan for the **IMes^{Me}-III** to **IMes^{Me}-IV** stage.

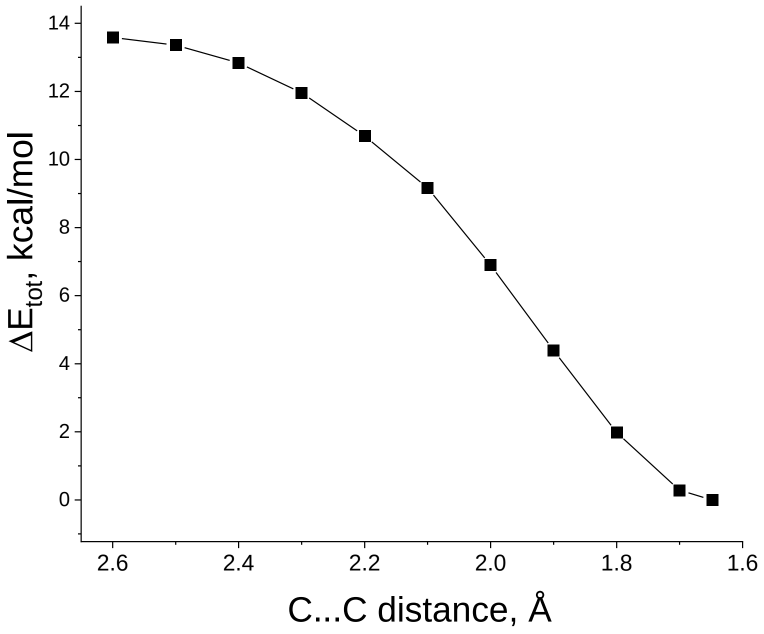

Figure S7. PES scan for the **IMes^{Me}-V** to **VI...IMes-I(H)** stage.

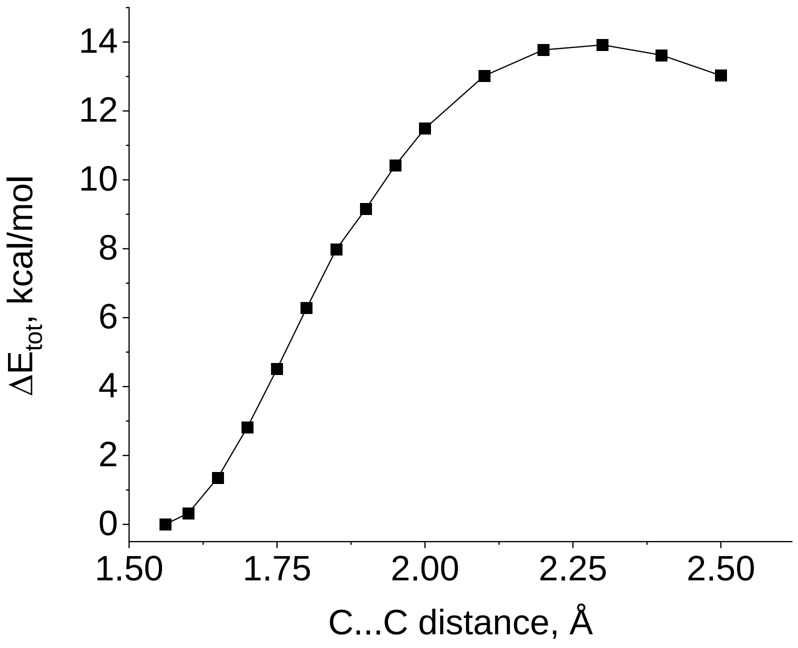

3.2. Optimized *xyz*-Cartesian coordinates

$[IMes^{Me}H]^+$ Etot = -1002.476003950496 a.u.

C	-0.007820000	0.000581000	-0.112038000
C	0.688827000	0.002005000	1.981084000
C	-0.677043000	0.002308000	1.992549000
N	1.076799000	0.000881000	0.652775000
N	-1.082944000	0.001501000	0.667121000
C	-2.437398000	0.000622000	0.183621000
C	-3.068489000	1.225259000	-0.033260000
C	-3.065822000	-1.225069000	-0.035887000
C	-4.377784000	1.194078000	-0.494840000
C	-4.375153000	-1.195825000	-0.497232000
C	-5.046824000	-0.001310000	-0.736207000
H	-4.890999000	2.134261000	-0.667784000
H	-4.886352000	-2.136789000	-0.671991000
C	2.430766000	-0.000018000	0.168356000
C	3.060769000	-1.225466000	-0.046112000
C	3.061858000	1.224777000	-0.047513000
C	4.374881000	-1.195991000	-0.494031000
C	4.375800000	1.193728000	-0.495364000
C	5.048658000	-0.001606000	-0.726398000
H	4.888086000	-2.136864000	-0.663428000
H	4.889780000	2.134000000	-0.665863000
C	2.359186000	2.529031000	0.178242000
H	1.565782000	2.689101000	-0.557965000
H	1.897757000	2.585102000	1.167288000
H	3.058335000	3.359428000	0.089297000
C	2.357170000	-2.528948000	0.181205000
H	1.895539000	-2.583425000	1.170247000
H	1.563788000	-2.689445000	-0.554929000
H	3.055770000	-3.359933000	0.093440000
C	-2.373928000	2.528553000	0.219756000
H	-2.010753000	2.604505000	1.248079000
H	-1.511284000	2.662368000	-0.439240000
H	-3.052337000	3.362440000	0.045185000
C	-2.368383000	-2.527321000	0.214670000
H	-1.507757000	-2.659689000	-0.447264000
H	-2.001614000	-2.602879000	1.241762000
H	-3.046040000	-3.362242000	0.042116000
C	-6.447839000	-0.002636000	-1.261813000
H	-6.444681000	-0.014774000	-2.355885000
H	-6.998699000	-0.882619000	-0.927584000
H	-6.993434000	0.887529000	-0.946732000
C	6.455890000	-0.002043000	-1.235316000

H	6.466005000	0.013621000	-2.329297000
H	7.004832000	0.875517000	-0.891646000
H	6.995597000	-0.894459000	-0.916502000
H	-0.015539000	-0.000492000	-1.189382000
C	-1.631266000	0.004881000	3.127727000
H	-2.658256000	-0.033995000	2.764673000
H	-1.470301000	-0.856040000	3.779484000
H	-1.522008000	0.906540000	3.734053000
C	1.658915000	0.002872000	3.098107000
H	1.128819000	-0.001075000	4.049497000
H	2.308931000	-0.874646000	3.065228000
H	2.302308000	0.885453000	3.069595000

$IMes^{Me}$-I(H) Etot = -1117.631512787924 a.u.

C	-1.776416000	0.521365000	-0.171676000
C	-2.521682000	2.239627000	-1.485649000
C	-1.211233000	2.050537000	-1.776940000
N	-2.831590000	1.299127000	-0.506485000
N	-0.789111000	1.004371000	-0.960465000
C	0.542534000	0.490792000	-0.952184000
C	1.499532000	1.111940000	-0.147508000
C	0.853605000	-0.616006000	-1.739276000
C	2.794688000	0.612926000	-0.163386000
C	2.165907000	-1.079264000	-1.722530000
C	3.149499000	-0.478119000	-0.949897000
H	3.545442000	1.085379000	0.463338000
H	2.420427000	-1.942647000	-2.329742000
C	-4.123966000	1.162700000	0.083598000
C	-5.019784000	0.231238000	-0.440596000
C	-4.457949000	1.970886000	1.170253000
C	-6.279212000	0.139903000	0.141519000
C	-5.727991000	1.842389000	1.718771000
C	-6.652953000	0.934146000	1.217979000
H	-6.987227000	-0.578761000	-0.260379000
H	-5.998965000	2.467861000	2.564147000
C	-3.465429000	2.930282000	1.751982000
H	-2.578732000	2.399480000	2.108491000
H	-3.119459000	3.659665000	1.014782000
H	-3.901153000	3.475341000	2.589712000
C	-4.631818000	-0.673115000	-1.568389000
H	-4.132343000	-0.130197000	-2.374179000
H	-3.935467000	-1.439682000	-1.213671000
H	-5.510295000	-1.170551000	-1.981117000
C	1.131189000	2.262621000	0.737886000
H	0.734364000	3.107359000	0.168406000

H	0.353601000	1.969762000	1.448213000
H	1.998819000	2.610276000	1.299221000
C	-0.193912000	-1.319628000	-2.544476000
H	-0.853832000	-1.893430000	-1.886096000
H	-0.821120000	-0.618648000	-3.100029000
H	0.265746000	-2.007928000	-3.254748000
C	4.561511000	-0.981492000	-0.965973000
H	4.975219000	-1.035542000	0.043585000
H	4.623939000	-1.974924000	-1.412334000
H	5.208506000	-0.316025000	-1.545637000
C	-8.007068000	0.790241000	1.844885000
H	-7.998746000	0.011612000	2.613990000
H	-8.326077000	1.718255000	2.322687000
H	-8.760001000	0.509232000	1.106118000
H	-1.927694000	-1.300776000	0.311151000
O	-2.082546000	-2.280109000	0.308928000
C	-1.935496000	-2.764347000	1.613639000
H	-2.126916000	-3.840432000	1.603395000
H	-0.921574000	-2.607956000	2.007993000
H	-2.645654000	-2.304043000	2.314846000
C	-0.320880000	2.724430000	-2.751457000
H	0.541633000	3.186358000	-2.263018000
H	0.070578000	2.020317000	-3.491103000
H	-0.865346000	3.504721000	-3.283486000
C	-3.517647000	3.185836000	-2.041792000
H	-3.057900000	3.812550000	-2.806097000
H	-4.359876000	2.658151000	-2.498058000
H	-3.931449000	3.842161000	-1.271066000

IMes^{Me}-TS1 Etot = -1460.730495766639 a.u.

C	-2.126372000	2.238476000	1.120578000
C	-1.294351000	3.080325000	0.460829000
N	-1.709169000	0.951480000	0.786059000
C	-0.659206000	0.961095000	-0.059289000
N	-0.408685000	2.270499000	-0.248200000
H	2.182601000	-0.585308000	1.424993000
O	2.293916000	-0.682164000	2.399698000
C	0.686252000	-0.745158000	-0.512615000
O	1.834026000	-0.389487000	-0.221395000
C	0.606512000	2.789337000	-1.111934000
C	1.859547000	3.097793000	-0.584322000
C	0.296293000	3.003198000	-2.453782000
C	2.810399000	3.631621000	-1.445632000
C	1.280980000	3.539088000	-3.274417000
C	2.544194000	3.855281000	-2.790577000

H	3.791876000	3.874379000	-1.049822000
H	1.051965000	3.712928000	-4.321633000
C	-2.347586000	-0.239015000	1.251885000
C	-3.341928000	-0.815364000	0.458920000
C	-1.982025000	-0.777750000	2.485782000
C	-3.960853000	-1.969468000	0.921094000
C	-2.636528000	-1.932236000	2.905604000
C	-3.618397000	-2.545741000	2.139561000
H	-4.737392000	-2.425950000	0.314566000
H	-2.364925000	-2.361887000	3.865068000
C	3.603693000	4.397021000	-3.701909000
H	4.233865000	3.588857000	-4.086024000
H	3.167408000	4.909558000	-4.561103000
H	4.257757000	5.098410000	-3.180306000
C	2.185927000	2.843087000	0.853099000
H	1.434982000	3.265079000	1.525777000
H	2.229685000	1.769826000	1.053189000
H	3.153069000	3.276488000	1.109179000
C	-0.910197000	-0.165028000	3.332141000
H	0.090787000	-0.440359000	2.981234000
H	-0.956795000	0.925547000	3.328148000
H	-1.001372000	-0.507249000	4.364025000
C	-3.731082000	-0.209381000	-0.854689000
H	-4.028768000	0.836823000	-0.743830000
H	-2.895620000	-0.227829000	-1.559530000
H	-4.565215000	-0.754597000	-1.297241000
C	-4.278828000	-3.808820000	2.603349000
H	-3.742941000	-4.686820000	2.229506000
H	-4.291940000	-3.874546000	3.692582000
H	-5.307048000	-3.876873000	2.243190000
C	-1.051237000	2.651712000	-3.005432000
H	-1.210285000	1.570065000	-2.988603000
H	-1.860832000	3.101047000	-2.424212000
H	-1.144415000	2.991388000	-4.037382000
H	0.069000000	-1.326249000	0.190907000
C	0.288132000	-0.985171000	-1.909605000
C	-0.643944000	-1.821024000	-2.444805000
O	0.973750000	-0.368391000	-2.896399000
C	-0.526259000	-1.699050000	-3.857967000
H	-1.320131000	-2.453048000	-1.890377000
C	0.470058000	-0.803499000	-4.067670000
H	-1.094829000	-2.214589000	-4.615512000
H	0.921542000	-0.396303000	-4.957357000
C	3.397829000	-1.507029000	2.649172000
H	4.332815000	-1.083912000	2.257443000
H	3.506750000	-1.614426000	3.731348000

H	3.277513000	-2.511860000	2.221342000
C	-1.239081000	4.560028000	0.414150000
H	-2.028105000	4.986325000	1.033572000
H	-0.280411000	4.939309000	0.777824000
H	-1.365915000	4.935887000	-0.604791000
C	-3.272278000	2.502348000	2.021817000
H	-4.193677000	2.050758000	1.643632000
H	-3.103305000	2.096364000	3.022836000
H	-3.436727000	3.575564000	2.117010000

IMes^{Me}-II Etot = -1460.758821695180 a.u.

C	-0.526325000	-0.474165000	-0.148083000
C	-1.226153000	-2.326322000	-1.195087000
C	0.105643000	-2.452903000	-0.974523000
N	-1.592626000	-1.087574000	-0.680473000
N	0.519220000	-1.291655000	-0.331719000
C	1.880103000	-1.045335000	0.049772000
C	2.724975000	-0.451715000	-0.894308000
C	2.329353000	-1.453673000	1.301307000
C	4.045527000	-0.238355000	-0.537920000
C	3.666278000	-1.206700000	1.609511000
C	4.532070000	-0.597764000	0.716943000
H	4.712267000	0.229398000	-1.255847000
H	4.029278000	-1.499151000	2.589824000
C	-2.932829000	-0.581989000	-0.687487000
C	-3.767752000	-0.885104000	0.388389000
C	-3.360410000	0.169036000	-1.781039000
C	-5.075114000	-0.416975000	0.333586000
C	-4.677177000	0.611670000	-1.785537000
C	-5.547668000	0.329763000	-0.739181000
H	-5.740580000	-0.638970000	1.162008000
H	-5.028577000	1.198371000	-2.628674000
C	-2.425405000	0.522351000	-2.896672000
H	-1.638103000	1.196758000	-2.546286000
H	-1.929922000	-0.358749000	-3.312689000
H	-2.962249000	1.021799000	-3.703233000
C	-3.261880000	-1.651164000	1.569710000
H	-2.913262000	-2.648488000	1.283423000
H	-2.413261000	-1.122835000	2.024355000
H	-4.050069000	-1.772048000	2.312957000
C	2.225190000	-0.053720000	-2.249467000
H	1.808531000	-0.904902000	-2.796299000
H	1.438606000	0.700355000	-2.176613000
H	3.036870000	0.362342000	-2.846304000
C	1.460330000	-2.135989000	2.309953000

H	1.375484000	-1.512345000	3.203689000
H	0.449251000	-2.305663000	1.946423000
H	1.904165000	-3.093212000	2.599593000
C	5.954217000	-0.311276000	1.091873000
H	6.082340000	0.747087000	1.337999000
H	6.260934000	-0.891701000	1.962960000
H	6.636844000	-0.538058000	0.269766000
C	-6.953246000	0.849192000	-0.750560000
H	-7.007121000	1.827126000	-0.262355000
H	-7.325444000	0.971300000	-1.769162000
H	-7.629351000	0.180379000	-0.215251000
C	-0.630220000	0.751539000	0.752980000
O	-0.644659000	0.270902000	2.003077000
H	0.349457000	0.558444000	3.220326000
C	0.416821000	1.790505000	0.452221000
C	1.415176000	2.331615000	1.189095000
O	0.329375000	2.423205000	-0.753816000
C	1.992738000	3.359578000	0.383056000
H	1.690650000	2.023200000	2.186636000
C	1.299166000	3.370808000	-0.777449000
H	2.812910000	4.010931000	0.641322000
H	1.361339000	3.964647000	-1.674559000
O	0.960886000	0.672188000	4.004608000
C	0.268260000	1.343405000	5.014509000
H	0.932067000	1.458925000	5.876728000
H	-0.057843000	2.347633000	4.706078000
H	-0.623734000	0.794497000	5.349400000
H	-1.590173000	1.224213000	0.437214000
C	-2.204533000	-3.236920000	-1.831876000
H	-1.706435000	-4.146076000	-2.166376000
H	-2.999621000	-3.519982000	-1.137260000
H	-2.682891000	-2.772591000	-2.698260000
C	1.044328000	-3.554734000	-1.284059000
H	1.476929000	-3.972465000	-0.371467000
H	0.524592000	-4.352986000	-1.812545000
H	1.874000000	-3.213423000	-1.907925000

IMes^{Me}-TS2 Etot = -1460.725189471182 a.u.

C	-0.400885000	-0.324617000	0.077970000
C	-1.209026000	-2.308142000	-0.589454000
C	0.139849000	-2.385109000	-0.634672000
N	-1.529816000	-1.022470000	-0.150949000
N	0.625294000	-1.150378000	-0.214151000
C	2.009824000	-0.788255000	-0.275375000
C	2.849581000	-1.122713000	0.787474000

C	2.483331000	-0.178228000	-1.439551000
C	4.193773000	-0.790510000	0.679009000
C	3.836876000	0.134743000	-1.491857000
C	4.703155000	-0.151481000	-0.444922000
H	4.859986000	-1.040335000	1.498953000
H	4.223007000	0.612553000	-2.386819000
C	-2.898006000	-0.599963000	-0.032991000
C	-3.553661000	-0.777833000	1.181507000
C	-3.545035000	-0.113555000	-1.167921000
C	-4.900687000	-0.447862000	1.247657000
C	-4.893465000	0.202917000	-1.047796000
C	-5.587144000	0.047158000	0.145601000
H	-5.424757000	-0.576243000	2.189899000
H	-5.413850000	0.588171000	-1.919318000
C	-2.816517000	0.110020000	-2.455076000
H	-2.116059000	0.953920000	-2.369616000
H	-2.233230000	-0.765968000	-2.753386000
H	-3.522439000	0.331831000	-3.256290000
C	-2.823481000	-1.258015000	2.396451000
H	-2.216951000	-2.144572000	2.194260000
H	-2.148426000	-0.478963000	2.762475000
H	-3.522716000	-1.499274000	3.197389000
C	2.334742000	-1.809666000	2.013938000
H	1.677122000	-1.144068000	2.578266000
H	1.760044000	-2.707596000	1.774429000
H	3.160722000	-2.099863000	2.663518000
C	1.585942000	0.121002000	-2.599202000
H	0.995204000	-0.754936000	-2.883437000
H	0.872640000	0.928899000	-2.379843000
H	2.178206000	0.419828000	-3.464548000
C	6.149441000	0.233329000	-0.517744000
H	6.778632000	-0.472367000	0.027867000
H	6.498594000	0.278323000	-1.550622000
H	6.308275000	1.221889000	-0.075710000
C	-7.031706000	0.432533000	0.250251000
H	-7.131379000	1.480176000	0.550653000
H	-7.545309000	0.317480000	-0.705882000
H	-7.553333000	-0.171551000	0.994906000
C	-0.282899000	1.102216000	0.403328000
O	-1.540938000	1.686445000	0.644935000
C	0.705998000	1.444916000	1.453717000
H	-1.640493000	2.235290000	-0.168285000
C	0.734725000	1.264553000	2.801998000
O	1.765151000	2.213413000	1.084798000
C	1.883002000	1.951989000	3.286748000
H	0.001828000	0.727508000	3.383531000

C	2.466987000	2.503261000	2.195478000
H	2.224422000	2.032052000	4.306616000
H	3.349237000	3.105790000	2.056015000
H	-0.041991000	1.664325000	-0.654488000
O	-0.627391000	2.406669000	-1.733365000
C	-0.085657000	3.645287000	-2.007494000
H	0.395811000	4.114821000	-1.129077000
H	0.689870000	3.606837000	-2.796449000
H	-0.847386000	4.362963000	-2.359949000
C	-2.250692000	-3.311171000	-0.907161000
H	-2.906265000	-2.969206000	-1.711613000
H	-1.784055000	-4.244811000	-1.219190000
H	-2.885704000	-3.518565000	-0.041958000
C	1.040619000	-3.478755000	-1.063705000
H	1.709239000	-3.797776000	-0.260383000
H	0.454998000	-4.340989000	-1.379922000
H	1.669759000	-3.167684000	-1.901762000

IMes^{Me}-III Etot = -1460.745928038013

C	-0.392706000	-0.351043000	-0.121297000
C	-1.168120000	-2.306287000	-0.990800000
C	0.169179000	-2.315348000	-1.122925000
N	-1.526216000	-1.102840000	-0.366278000
N	0.659726000	-1.103322000	-0.603456000
C	2.035829000	-0.932553000	-0.277628000
C	2.927974000	-0.538406000	-1.277516000
C	2.473936000	-1.230995000	1.014460000
C	4.274977000	-0.440527000	-0.955017000
C	3.830734000	-1.103024000	1.291790000
C	4.745438000	-0.707228000	0.325467000
H	4.976477000	-0.137337000	-1.726754000
H	4.181062000	-1.335220000	2.293134000
C	-2.863891000	-0.612340000	-0.368042000
C	-3.739133000	-0.985619000	0.653268000
C	-3.283714000	0.194975000	-1.428153000
C	-5.050410000	-0.527322000	0.591228000
C	-4.600567000	0.639759000	-1.438844000
C	-5.499187000	0.291102000	-0.437668000
H	-5.740153000	-0.817468000	1.378363000
H	-4.935104000	1.264344000	-2.262073000
C	-2.342532000	0.567526000	-2.532976000
H	-1.523464000	1.190869000	-2.163309000
H	-1.884325000	-0.317565000	-2.983110000
H	-2.865620000	1.116898000	-3.316235000
C	-3.286471000	-1.817234000	1.812599000

H	-2.605854000	-2.613976000	1.509932000
H	-2.749067000	-1.206428000	2.543741000
H	-4.141731000	-2.266997000	2.319005000
C	2.452131000	-0.187167000	-2.653538000
H	1.901707000	-1.002842000	-3.126889000
H	1.777715000	0.670935000	-2.612576000
H	3.295250000	0.067480000	-3.296972000
C	1.524367000	-1.696996000	2.073650000
H	0.876698000	-0.888650000	2.423414000
H	0.866456000	-2.486971000	1.701805000
H	2.070920000	-2.085630000	2.933500000
C	6.198270000	-0.550048000	0.658739000
H	6.417059000	0.478322000	0.963014000
H	6.491039000	-1.203065000	1.482821000
H	6.831987000	-0.777676000	-0.200601000
C	-6.909354000	0.799484000	-0.457566000
H	-6.991634000	1.743487000	0.090285000
H	-7.252440000	0.983636000	-1.477279000
H	-7.594601000	0.091045000	0.011440000
C	-0.374052000	0.867997000	0.520454000
O	-1.482172000	1.148334000	1.327441000
H	-1.303821000	0.624036000	3.086863000
C	0.630517000	1.881449000	0.552912000
C	0.798081000	2.936679000	1.416737000
O	1.537377000	1.982644000	-0.454367000
C	1.879703000	3.708285000	0.905748000
H	0.231863000	3.116361000	2.315618000
C	2.298072000	3.085336000	-0.217999000
H	2.299322000	4.607179000	1.329415000
H	3.089694000	3.272202000	-0.923536000
O	-1.198184000	0.180039000	3.943317000
C	-0.616191000	1.075325000	4.854331000
H	-0.504481000	0.554964000	5.807478000
H	0.378219000	1.411941000	4.533044000
H	-1.241166000	1.962056000	5.027341000
H	-1.932187000	1.934247000	0.997835000
C	-2.188108000	-3.296055000	-1.405369000
H	-1.708525000	-4.146375000	-1.888896000
H	-2.765052000	-3.670046000	-0.554944000
H	-2.904256000	-2.864459000	-2.110966000
C	1.077744000	-3.375793000	-1.615101000
H	1.782800000	-3.689087000	-0.839576000
H	0.498534000	-4.247772000	-1.917135000
H	1.672130000	-3.052265000	-2.473436000

IMes^{Me}-IV Etot = -1803.861918482877 a.u.

C	0.578519000	-1.544947000	-0.379160000
C	-0.494965000	0.425033000	-0.321087000
C	0.790662000	0.647413000	0.039498000
N	-0.613679000	-0.937109000	-0.573410000
N	1.435171000	-0.579705000	0.002564000
C	2.801684000	-0.679604000	0.457738000
C	3.842873000	-0.449267000	-0.432436000
C	3.013013000	-0.870334000	1.824931000
C	5.138578000	-0.456315000	0.077013000
C	4.322723000	-0.868593000	2.278397000
C	5.401109000	-0.673802000	1.420602000
H	5.964515000	-0.285763000	-0.606556000
H	4.505454000	-1.030580000	3.336203000
C	-1.933352000	-1.495444000	-0.763701000
C	-2.510738000	-1.541310000	-2.028218000
C	-2.652003000	-1.836758000	0.392824000
C	-3.812097000	-2.030414000	-2.128432000
C	-3.944706000	-2.308930000	0.232864000
C	-4.537082000	-2.434766000	-1.020452000
H	-4.268254000	-2.083324000	-3.112048000
H	-4.503502000	-2.594040000	1.118453000
C	-2.047742000	-1.731344000	1.756897000
H	-1.250592000	-2.477074000	1.892419000
H	-1.609541000	-0.744155000	1.931997000
H	-2.808021000	-1.901805000	2.519034000
C	-1.818376000	-1.084633000	-3.274239000
H	-1.063821000	-0.322049000	-3.076772000
H	-1.319697000	-1.920549000	-3.769775000
H	-2.545205000	-0.667972000	-3.973397000
C	3.615619000	-0.223668000	-1.894242000
H	3.380892000	-1.163277000	-2.400729000
H	2.782522000	0.457992000	-2.084759000
H	4.508322000	0.197487000	-2.357527000
C	1.873770000	-1.095434000	2.768483000
H	1.179394000	-0.248855000	2.774362000
H	1.296387000	-1.984185000	2.493286000
H	2.242162000	-1.228410000	3.785594000
C	6.806616000	-0.717563000	1.937522000
H	6.890927000	-0.221114000	2.906529000
H	7.135113000	-1.752344000	2.073577000
H	7.501469000	-0.239297000	1.245622000
C	-5.918572000	-2.995398000	-1.158157000
H	-5.903428000	-4.082844000	-1.040093000
H	-6.587765000	-2.596663000	-0.392778000
H	-6.344489000	-2.773757000	-2.137574000

C	0.994521000	-3.014704000	-0.563044000
O	2.133902000	-3.241588000	0.185027000
C	1.297022000	-3.257630000	-2.006687000
C	-0.108543000	-3.979899000	0.141319000
H	1.674744000	-3.626713000	1.018263000
C	1.227670000	-2.503498000	-3.136530000
O	1.835901000	-4.458995000	-2.301962000
O	0.226104000	-3.948073000	1.451863000
H	-1.094243000	-3.555442000	-0.088368000
C	-0.120960000	-5.358953000	-0.435504000
C	1.739655000	-3.308575000	-4.196637000
H	0.883195000	-1.486619000	-3.207869000
C	2.091460000	-4.482665000	-3.623217000
C	0.336369000	-6.549579000	0.021236000
O	-0.756002000	-5.528694000	-1.626018000
H	1.838079000	-3.044472000	-5.237402000
H	2.520033000	-5.394981000	-4.003223000
C	-0.024116000	-7.518104000	-0.962259000
H	0.836791000	-6.710240000	0.962463000
C	-0.678242000	-6.843359000	-1.935979000
H	0.169401000	-8.579076000	-0.939643000
H	-1.141362000	-7.140901000	-2.862420000
H	0.091102000	-5.170437000	2.623437000
O	0.149197000	-5.816843000	3.366834000
C	0.978896000	-5.270046000	4.345402000
H	1.991590000	-5.052076000	3.972942000
H	0.575259000	-4.339181000	4.773847000
H	1.075505000	-5.992659000	5.161013000
C	-1.642259000	1.355188000	-0.429316000
H	-2.059503000	1.366720000	-1.438852000
H	-2.449749000	1.074726000	0.251104000
H	-1.322025000	2.366387000	-0.181997000
C	1.482323000	1.895619000	0.435142000
H	1.873718000	1.828860000	1.452857000
H	2.328787000	2.110925000	-0.220819000
H	0.789632000	2.734760000	0.387980000

IMes^{Me}-V Etot= -1803.865982857098 a.u.

C	0.335663000	-1.423905000	-0.141960000
C	-0.695210000	0.574924000	-0.087244000
C	0.624035000	0.789983000	0.119664000
N	-0.854778000	-0.799142000	-0.247594000
N	1.242097000	-0.448751000	0.075601000
C	2.664357000	-0.573062000	0.250039000
C	3.485524000	-0.393589000	-0.864021000

C	3.177820000	-0.735790000	1.537931000
C	4.859213000	-0.475762000	-0.672641000
C	4.558023000	-0.814832000	1.668996000
C	5.412796000	-0.704863000	0.580183000
H	5.511778000	-0.358361000	-1.532195000
H	4.970705000	-0.980492000	2.658510000
C	-2.167989000	-1.332712000	-0.480569000
C	-2.619485000	-1.447040000	-1.796396000
C	-2.985924000	-1.602482000	0.613707000
C	-3.921174000	-1.882585000	-1.999377000
C	-4.280850000	-2.036740000	0.354649000
C	-4.763876000	-2.191851000	-0.936855000
H	-4.284814000	-1.985817000	-3.017007000
H	-4.926430000	-2.270073000	1.195354000
C	-2.521056000	-1.404089000	2.022146000
H	-1.471141000	-1.666423000	2.145560000
H	-2.649133000	-0.361982000	2.335275000
H	-3.104305000	-2.025308000	2.701536000
C	-1.730186000	-1.139546000	-2.961817000
H	-1.202286000	-0.190423000	-2.841263000
H	-0.969024000	-1.914720000	-3.090669000
H	-2.310209000	-1.092573000	-3.883448000
C	2.932626000	-0.073755000	-2.218982000
H	3.711889000	-0.159882000	-2.976229000
H	2.122867000	-0.749332000	-2.494464000
H	2.543138000	0.948835000	-2.260347000
C	2.297464000	-0.783267000	2.745732000
H	1.887248000	0.207954000	2.970257000
H	1.472893000	-1.479802000	2.587717000
H	2.864259000	-1.120788000	3.612268000
C	6.894038000	-0.846530000	0.754796000
H	7.236368000	-0.354924000	1.667707000
H	7.172228000	-1.901946000	0.833177000
H	7.439146000	-0.421661000	-0.089663000
C	-6.148882000	-2.706311000	-1.184056000
H	-6.136052000	-3.792066000	-1.319433000
H	-6.810748000	-2.489055000	-0.344325000
H	-6.582013000	-2.269971000	-2.086091000
C	0.686557000	-2.939943000	-0.016063000
O	0.992192000	-3.126625000	1.274578000
C	1.804067000	-3.260998000	-0.990147000
C	-0.567603000	-3.884270000	-0.350416000
H	-0.534957000	-3.784569000	1.490513000
C	1.881480000	-3.305779000	-2.348246000
O	2.968054000	-3.683356000	-0.457432000
O	-1.268985000	-3.988569000	0.846855000

H	-1.200803000	-3.442494000	-1.124788000
C	-0.151831000	-5.225709000	-0.855010000
C	3.186731000	-3.779755000	-2.664284000
H	1.093012000	-3.069963000	-3.045210000
C	3.796431000	-3.991770000	-1.473993000
C	0.444872000	-6.298034000	-0.278167000
O	-0.429279000	-5.498797000	-2.156636000
H	3.606652000	-3.952839000	-3.642471000
H	4.773993000	-4.347407000	-1.194490000
C	0.552093000	-7.293694000	-1.292824000
H	0.767907000	-6.361593000	0.748066000
C	0.008338000	-6.753536000	-2.407832000
H	0.974358000	-8.281963000	-1.201761000
H	-0.144712000	-7.118735000	-3.409712000
H	2.383625000	-3.684555000	2.171732000
O	3.076804000	-3.948546000	2.814851000
C	3.242577000	-5.332884000	2.740376000
H	2.336939000	-5.883942000	3.035930000
H	3.527007000	-5.670822000	1.733539000
H	4.041967000	-5.620957000	3.429016000
C	-1.831151000	1.521991000	-0.164102000
H	-2.333725000	1.472145000	-1.133249000
H	-2.584539000	1.316095000	0.599566000
H	-1.471289000	2.540097000	-0.020301000
C	1.373828000	2.040936000	0.375149000
H	1.895863000	1.999817000	1.333697000
H	2.127592000	2.226913000	-0.393083000
H	0.689604000	2.888243000	0.394383000

VI...IMes^{Me}-I(H) Etot = -1803.867930628404 a.u.

C	-1.845449000	0.682538000	-0.182153000
C	-2.649840000	2.097433000	-1.783842000
C	-1.332377000	1.908407000	-2.041202000
N	-2.927482000	1.344821000	-0.645456000
N	-0.873310000	1.047819000	-1.047558000
C	0.472822000	0.588324000	-0.941430000
C	1.412437000	1.380880000	-0.280517000
C	0.809040000	-0.650436000	-1.488413000
C	2.716852000	0.906581000	-0.193567000
C	2.125169000	-1.082478000	-1.371576000
C	3.093821000	-0.316419000	-0.733962000
H	3.456776000	1.510967000	0.322505000
H	2.397988000	-2.046195000	-1.791436000
C	-4.215122000	1.277571000	-0.031366000
C	-5.047365000	0.197827000	-0.319458000

C	-4.605409000	2.297818000	0.838283000
C	-6.304894000	0.164944000	0.273590000
C	-5.872605000	2.221176000	1.402158000
C	-6.735981000	1.165125000	1.134011000
H	-6.963157000	-0.670538000	0.055192000
H	-6.189444000	3.009545000	2.078619000
C	-3.681538000	3.422286000	1.190859000
H	-2.848721000	3.073950000	1.809684000
H	-3.242542000	3.888934000	0.305899000
H	-4.215010000	4.191820000	1.750251000
C	-4.597537000	-0.916326000	-1.213468000
H	-4.149265000	-0.543070000	-2.137359000
H	-3.841046000	-1.527320000	-0.712593000
H	-5.437318000	-1.561217000	-1.474777000
C	1.030481000	2.684423000	0.349805000
H	0.484739000	3.328789000	-0.343398000
H	0.380374000	2.539091000	1.217854000
H	1.918988000	3.220957000	0.684517000
C	-0.223113000	-1.506376000	-2.156122000
H	-0.966960000	-1.852569000	-1.432931000
H	-0.765656000	-0.956321000	-2.929185000
H	0.240283000	-2.378788000	-2.617794000
C	4.514870000	-0.787281000	-0.652311000
H	5.011922000	-0.398106000	0.238259000
H	4.573260000	-1.877156000	-0.629435000
H	5.088965000	-0.448805000	-1.520570000
C	-8.086307000	1.098434000	1.781749000
H	-8.001827000	0.784288000	2.826511000
H	-8.579391000	2.073139000	1.775994000
H	-8.736518000	0.384358000	1.274024000
H	-1.871803000	-0.739547000	0.938603000
O	-1.909972000	-1.642721000	1.390098000
C	-0.727760000	-1.873065000	2.061461000
C	-0.392710000	-0.725852000	3.036373000
H	0.135750000	-1.979299000	1.383093000
C	-0.846759000	-3.143771000	2.839199000
O	-1.240474000	0.067332000	3.385095000
C	0.983444000	-0.635210000	3.512670000
C	-1.872653000	-3.687534000	3.538081000
O	0.241421000	-3.949941000	2.903655000
C	2.127124000	-1.324393000	3.222121000
O	1.258041000	0.332839000	4.426660000
C	-1.384209000	-4.917289000	4.069309000
H	-2.855594000	-3.258210000	3.640262000
C	-0.099990000	-5.021998000	3.656937000
C	3.156061000	-0.745969000	4.003457000

H	2.213910000	-2.150005000	2.535396000
C	2.562784000	0.252534000	4.709729000
H	-1.920535000	-5.629673000	4.675772000
H	0.667711000	-5.763998000	3.799549000
H	4.195418000	-1.029638000	4.040506000
H	2.936727000	0.963390000	5.428956000
O	-0.910612000	2.848838000	3.383143000
C	-0.772330000	3.428598000	4.653177000
H	-1.039634000	1.892400000	3.492301000
H	-1.672534000	3.303714000	5.269046000
H	0.080473000	3.016127000	5.207852000
H	-0.602801000	4.498643000	4.515859000
C	-3.681427000	2.879214000	-2.505113000
H	-3.246007000	3.361517000	-3.380413000
H	-4.502599000	2.240701000	-2.842575000
H	-4.120200000	3.656644000	-1.873970000
C	-0.464344000	2.425103000	-3.125242000
H	0.364481000	3.021993000	-2.734383000
H	-0.024948000	1.612516000	-3.710822000
H	-1.041256000	3.054846000	-3.802599000

$[IPr^{Me}H]^+$ Etot= -1238.129443953881 a.u. symmetry c1

C	-1.837939000	0.562542000	-0.315826000
C	-2.594852000	2.158519000	-1.647144000
C	-1.274785000	1.963908000	-1.931992000
N	-2.915311000	1.268335000	-0.633517000
N	-0.831898000	0.960894000	-1.083243000
C	0.501099000	0.424789000	-1.023576000
C	1.418641000	1.025240000	-0.155344000
C	0.812508000	-0.672749000	-1.832750000
C	2.697312000	0.477900000	-0.107135000
C	2.106167000	-1.178290000	-1.743954000
C	3.037973000	-0.610800000	-0.891550000
H	3.435038000	0.910223000	0.559127000
H	2.385114000	-2.032858000	-2.349681000
C	-4.203346000	1.116322000	-0.012468000
C	-5.104292000	0.202843000	-0.569392000
C	-4.489937000	1.886420000	1.119411000
C	-6.338659000	0.066774000	0.058769000
C	-5.739663000	1.709587000	1.705966000
C	-6.653600000	0.810694000	1.183095000
H	-7.061501000	-0.636746000	-0.338174000
H	-5.997605000	2.283130000	2.588868000
C	-3.500714000	2.856430000	1.727528000
H	-2.636114000	2.932862000	1.063582000

C	-4.775906000	-0.641053000	-1.781402000
H	-3.821460000	-0.301843000	-2.191534000
C	1.065286000	2.199792000	0.730402000
H	0.065148000	2.548223000	0.461259000
C	-0.194449000	-1.328279000	-2.752264000
H	-1.100300000	-0.717147000	-2.766033000
H	-1.787457000	-0.205404000	0.439089000
H	4.039746000	-1.022377000	-0.836965000
H	-7.620839000	0.687652000	1.657684000
C	-0.580878000	-2.711197000	-2.228004000
H	-1.340417000	-3.159649000	-2.873211000
H	0.286211000	-3.376959000	-2.212674000
H	-0.983432000	-2.654526000	-1.213873000
C	0.316560000	-1.413010000	-4.188687000
H	1.195076000	-2.058568000	-4.262254000
H	-0.459007000	-1.832615000	-4.833897000
H	0.585479000	-0.427216000	-4.575002000
C	2.022966000	3.372072000	0.529297000
H	1.702131000	4.221461000	1.137148000
H	3.040212000	3.111970000	0.831861000
H	2.049754000	3.690613000	-0.515308000
C	1.024382000	1.773986000	2.197842000
H	0.312796000	0.960236000	2.356558000
H	2.008722000	1.434456000	2.531145000
H	0.726279000	2.616934000	2.826205000
C	-3.000234000	2.339781000	3.076304000
H	-3.822297000	2.265102000	3.793221000
H	-2.543115000	1.352120000	2.979918000
H	-2.253799000	3.023689000	3.487877000
C	-4.090784000	4.258016000	1.866107000
H	-4.439169000	4.640926000	0.904193000
H	-4.933353000	4.268956000	2.561829000
H	-3.332207000	4.943247000	2.251975000
C	-5.822370000	-0.487804000	-2.882749000
H	-5.522366000	-1.063287000	-3.761786000
H	-6.798285000	-0.858535000	-2.560029000
H	-5.937072000	0.556689000	-3.181520000
C	-4.611220000	-2.108397000	-1.385874000
H	-5.546699000	-2.510256000	-0.987535000
H	-4.330942000	-2.704255000	-2.258037000
H	-3.837518000	-2.231420000	-0.624299000
C	-0.387206000	2.618895000	-2.917022000
H	0.452149000	3.115211000	-2.424228000
H	0.020855000	1.892987000	-3.624177000
H	-0.945492000	3.366410000	-3.478143000
C	-3.584940000	3.092239000	-2.225918000

H	-3.113110000	3.700659000	-2.995657000
H	-4.420082000	2.552692000	-2.678782000
H	-3.991467000	3.759574000	-1.462483000

IPr^{Me}-I(H) Etot -1353.286521911816 a.u.

C	-1.836155000	0.466305000	-0.239030000
C	-2.589776000	2.132131000	-1.610393000
C	-1.276894000	1.940023000	-1.894158000
N	-2.897069000	1.223409000	-0.601949000
N	-0.849688000	0.924640000	-1.043848000
C	0.486099000	0.415084000	-1.006479000
C	1.417106000	1.020373000	-0.152353000
C	0.818225000	-0.676009000	-1.819245000
C	2.703701000	0.489597000	-0.114073000
C	2.117860000	-1.170413000	-1.743822000
C	3.052596000	-0.596450000	-0.898931000
H	3.442590000	0.933387000	0.544222000
H	2.399937000	-2.020350000	-2.355490000
C	-4.190563000	1.098638000	-0.004599000
C	-5.110529000	0.201065000	-0.560623000
C	-4.494921000	1.878708000	1.118325000
C	-6.358972000	0.090744000	0.046076000
C	-5.756364000	1.731519000	1.688950000
C	-6.681120000	0.846904000	1.160201000
H	-7.087741000	-0.602392000	-0.359669000
H	-6.016037000	2.317434000	2.563814000
C	-3.501169000	2.840246000	1.733750000
H	-2.623019000	2.879749000	1.085525000
C	-4.783798000	-0.657918000	-1.762784000
H	-3.797219000	-0.365409000	-2.128677000
C	1.068416000	2.202003000	0.727523000
H	0.048018000	2.510462000	0.489860000
C	-0.180895000	-1.333546000	-2.746798000
H	-1.098899000	-0.742052000	-2.728912000
H	-1.828790000	-1.046872000	0.843395000
O	-1.868184000	-1.895773000	1.374694000
C	-1.107660000	-1.733254000	2.544863000
H	-1.221294000	-2.629309000	3.160390000
H	-0.037090000	-1.604334000	2.334548000
H	-1.439107000	-0.873091000	3.142059000
H	4.059491000	-0.997085000	-0.852662000
H	-7.658282000	0.745527000	1.619925000
C	-0.531099000	-2.740874000	-2.264472000
H	-1.273749000	-3.193235000	-2.927293000
H	0.353359000	-3.383955000	-2.261291000

H	-0.943695000	-2.720908000	-1.253323000
C	0.319038000	-1.365595000	-4.189778000
H	1.212413000	-1.987550000	-4.289145000
H	-0.451097000	-1.784703000	-4.842423000
H	0.562954000	-0.363682000	-4.550972000
C	1.986087000	3.394740000	0.465478000
H	1.669102000	4.250003000	1.067767000
H	3.021182000	3.166256000	0.732527000
H	1.966854000	3.692521000	-0.585451000
C	1.100025000	1.814733000	2.205301000
H	0.417279000	0.988522000	2.414452000
H	2.105568000	1.508778000	2.507264000
H	0.807123000	2.666601000	2.824749000
C	-3.040738000	2.341820000	3.103203000
H	-3.879338000	2.295888000	3.803687000
H	-2.601295000	1.343968000	3.034923000
H	-2.290203000	3.018350000	3.520621000
C	-4.063408000	4.256461000	1.836353000
H	-4.385587000	4.630547000	0.861741000
H	-4.919713000	4.297510000	2.514476000
H	-3.299227000	4.934142000	2.225481000
C	-5.780491000	-0.450116000	-2.901045000
H	-5.484341000	-1.042533000	-3.770570000
H	-6.786369000	-0.765221000	-2.611450000
H	-5.829035000	0.598447000	-3.204281000
C	-4.711737000	-2.133287000	-1.370314000
H	-5.687041000	-2.493362000	-1.030807000
H	-4.409339000	-2.739703000	-2.228186000
H	-3.992637000	-2.294353000	-0.564172000
C	-0.391030000	2.603366000	-2.879366000
H	0.450495000	3.103623000	-2.393143000
H	0.022040000	1.886249000	-3.593666000
H	-0.950446000	3.352717000	-3.439086000
C	-3.577093000	3.074661000	-2.186736000
H	-3.101789000	3.694480000	-2.946745000
H	-4.412529000	2.547030000	-2.654632000
H	-3.994102000	3.736456000	-1.423130000

IPr^{Me}-TS1 Etot = -1696.375536100965 a.u.

C	-2.311411000	2.294536000	1.047653000
C	-1.447723000	3.119518000	0.407562000
N	-1.878999000	0.998754000	0.774254000
C	-0.784520000	0.982287000	-0.018835000
N	-0.519481000	2.292680000	-0.224357000
H	2.683095000	-1.204072000	1.777732000

O	3.365344000	-1.572026000	2.369670000
C	0.600611000	-0.834608000	-0.087408000
O	1.603726000	-0.491531000	0.557236000
C	0.500356000	2.781587000	-1.106948000
C	1.747215000	3.161878000	-0.590407000
C	0.194527000	2.897420000	-2.471221000
C	2.708370000	3.617407000	-1.490005000
C	1.192536000	3.355295000	-3.326719000
C	2.441807000	3.705195000	-2.844917000
H	3.683283000	3.912267000	-1.118088000
H	0.983160000	3.445768000	-4.386931000
C	-2.576389000	-0.173814000	1.216091000
C	-3.603402000	-0.686230000	0.409854000
C	-2.236596000	-0.753536000	2.446277000
C	-4.265631000	-1.829232000	0.849285000
C	-2.933885000	-1.892635000	2.841049000
C	-3.933828000	-2.431361000	2.050590000
H	-5.057244000	-2.251432000	0.240143000
H	-2.687392000	-2.364034000	3.785943000
C	2.084327000	3.131928000	0.886070000
H	1.237881000	2.691963000	1.419183000
C	-1.163551000	-0.194173000	3.356361000
H	-0.707975000	0.664701000	2.858043000
C	-4.023185000	-0.045159000	-0.896525000
H	-3.428145000	0.859222000	-1.038263000
C	-1.172551000	2.577548000	-3.039382000
H	-1.819706000	2.266869000	-2.217085000
H	-0.240858000	-1.354067000	0.390093000
C	0.698503000	-1.138916000	-1.518205000
C	0.039014000	-2.051039000	-2.284252000
O	1.708759000	-0.583925000	-2.232348000
C	0.681445000	-2.049954000	-3.554249000
H	-0.791175000	-2.663399000	-1.967738000
C	1.681634000	-1.138786000	-3.463057000
H	0.434957000	-2.646840000	-4.418549000
H	2.425400000	-0.785745000	-4.159618000
C	4.410882000	-2.076684000	1.569163000
H	4.877359000	-1.294188000	0.957946000
H	5.173868000	-2.489596000	2.232029000
H	4.070303000	-2.877410000	0.901207000
H	3.207408000	4.059159000	-3.526796000
H	-4.461789000	-3.321355000	2.375415000
C	3.316909000	2.276544000	1.175182000
H	3.495706000	2.236548000	2.253111000
H	3.183397000	1.257983000	0.811396000
H	4.208417000	2.704120000	0.708795000

C	2.305608000	4.546307000	1.425522000
H	1.450728000	5.198135000	1.238092000
H	2.479422000	4.513028000	2.504137000
H	3.182815000	5.003171000	0.959194000
C	-1.756342000	0.288800000	4.680401000
H	-2.539518000	1.034215000	4.529026000
H	-2.189053000	-0.544725000	5.240204000
H	-0.974681000	0.737067000	5.299055000
C	-0.061726000	-1.217959000	3.622348000
H	-0.453353000	-2.085693000	4.159655000
H	0.396561000	-1.565267000	2.695391000
H	0.722246000	-0.770897000	4.238993000
C	-5.494415000	0.368859000	-0.871812000
H	-5.751179000	0.886271000	-1.799712000
H	-6.149125000	-0.502102000	-0.784176000
H	-5.712450000	1.039499000	-0.037759000
C	-3.753105000	-0.966861000	-2.083766000
H	-2.696624000	-1.232042000	-2.149272000
H	-4.332247000	-1.890743000	-2.002129000
H	-4.038998000	-0.472808000	-3.015985000
C	-1.114629000	1.426551000	-4.040561000
H	-0.488680000	1.683081000	-4.899625000
H	-0.711049000	0.520134000	-3.585864000
H	-2.117709000	1.203012000	-4.413326000
C	-1.806445000	3.811112000	-3.682675000
H	-1.226166000	4.148071000	-4.545717000
H	-2.815212000	3.574038000	-4.030601000
H	-1.876522000	4.642565000	-2.977752000
C	-1.423533000	4.598754000	0.328560000
H	-0.643123000	5.028734000	0.960662000
H	-1.245407000	4.941025000	-0.692768000
H	-2.379712000	5.003435000	0.660339000
C	-3.493003000	2.606537000	1.884619000
H	-4.375136000	2.051329000	1.559197000
H	-3.321516000	2.361563000	2.935628000
H	-3.719719000	3.670763000	1.823109000

IPr^{Me}-II Etot = -1696.394063474064 a.u.

C	-0.323380000000	-0.503751000000	0.037727000000
C	-1.180746000000	-2.224402000000	-1.105244000000
C	0.154264000000	-2.433109000000	-0.981530000000
N	-1.453511000000	-1.024767000000	-0.466978000000
N	0.667326000000	-1.354557000000	-0.273907000000
C	2.068622000000	-1.197973000000	0.017492000000
C	2.880529000000	-0.586307000000	-0.947943000000

C	2.578998000000	-1.741186000000	1.203656000000
C	4.240565000000	-0.485350000000	-0.669904000000
C	3.946070000000	-1.605771000000	1.430191000000
C	4.768332000000	-0.979682000000	0.509913000000
H	4.897011000000	-0.016588000000	-1.393726000000
H	4.374487000000	-2.011063000000	2.339634000000
C	-2.771087000000	-0.453669000000	-0.373505000000
C	-3.598229000000	-0.847382000000	0.686472000000
C	-3.196986000000	0.419138000000	-1.384502000000
C	-4.887161000000	-0.322401000000	0.721355000000
C	-4.496637000000	0.909705000000	-1.299313000000
C	-5.333636000000	0.546219000000	-0.258517000000
H	-5.552890000000	-0.605892000000	1.528559000000
H	-4.860419000000	1.584165000000	-2.065655000000
C	-2.327634000000	0.811248000000	-2.560925000000
H	-1.302393000000	0.495363000000	-2.353041000000
C	-3.166119000000	-1.821057000000	1.762263000000
H	-2.108655000000	-2.051896000000	1.614507000000
C	2.355025000000	-0.082521000000	-2.276530000000
H	1.263185000000	-0.063892000000	-2.231234000000
C	1.735424000000	-2.512403000000	2.195885000000
H	0.686782000000	-2.371851000000	1.929011000000
C	-0.300330000000	0.687966000000	0.987445000000
O	-0.045824000000	0.237934000000	2.235331000000
H	-0.482975000000	1.177944000000	3.381539000000
C	0.611172000000	1.819829000000	0.578782000000
C	1.685728000000	2.375570000000	1.193535000000
O	0.229042000000	2.601168000000	-0.466116000000
C	1.988636000000	3.574387000000	0.478422000000
H	2.182326000000	1.985536000000	2.067199000000
C	1.073462000000	3.661062000000	-0.514873000000
H	2.781581000000	4.278385000000	0.679805000000
H	0.899911000000	4.372205000000	-1.306634000000
O	-0.737309000000	1.749985000000	4.184030000000
C	-1.654792000000	2.724951000000	3.767926000000
H	-1.900465000000	3.368797000000	4.617775000000
H	-1.253056000000	3.367122000000	2.970712000000
H	-2.595278000000	2.288295000000	3.401016000000
H	-1.321385000000	1.111127000000	0.859062000000
C	1.906990000000	-2.003885000000	3.624099000000
H	1.246811000000	-2.561257000000	4.294088000000
H	2.930394000000	-2.143436000000	3.981791000000
H	1.646159000000	-0.947336000000	3.686366000000
C	2.060012000000	-4.006095000000	2.125947000000
H	1.409899000000	-4.562006000000	2.806624000000
H	1.923288000000	-4.408682000000	1.120695000000

H	3.095770000000	-4.191013000000	2.424555000000
C	-3.312197000000	-1.222738000000	3.160155000000
H	-4.357986000000	-1.005950000000	3.391921000000
H	-2.947275000000	-1.933589000000	3.905957000000
H	-2.736871000000	-0.300735000000	3.260040000000
C	-3.951930000000	-3.129107000000	1.662390000000
H	-3.591072000000	-3.839585000000	2.410370000000
H	-5.015762000000	-2.957687000000	1.847503000000
H	-3.850933000000	-3.589624000000	0.677877000000
C	-2.304643000000	2.321815000000	-2.783059000000
H	-3.283681000000	2.694952000000	-3.093115000000
H	-2.001972000000	2.853877000000	-1.880254000000
H	-1.593939000000	2.565458000000	-3.576732000000
C	-2.785161000000	0.097930000000	-3.834497000000
H	-2.766519000000	-0.987756000000	-3.722370000000
H	-3.805041000000	0.393850000000	-4.095496000000
H	-2.131833000000	0.365464000000	-4.668883000000
C	2.761626000000	-1.020166000000	-3.415397000000
H	3.850515000000	-1.055769000000	-3.510072000000
H	2.403029000000	-2.038540000000	-3.260918000000
H	2.353044000000	-0.656129000000	-4.361608000000
C	2.825799000000	1.335201000000	-2.588360000000
H	2.599006000000	2.025184000000	-1.775045000000
H	3.902954000000	1.365345000000	-2.770880000000
H	2.330340000000	1.696542000000	-3.493096000000
H	-6.342933000000	0.940348000000	-0.213417000000
H	5.830803000000	-0.888199000000	0.707594000000
C	-2.197181000000	-3.081188000000	-1.759764000000
H	-1.861579000000	-3.371357000000	-2.757689000000
H	-2.363192000000	-3.997595000000	-1.187336000000
H	-3.151675000000	-2.564888000000	-1.854218000000
C	0.953078000000	-3.584741000000	-1.462904000000
H	2.018663000000	-3.427478000000	-1.302480000000
H	0.662069000000	-4.499591000000	-0.940558000000
H	0.784646000000	-3.747465000000	-2.529835000000

IPr^{Me}-TS2 Etot = -1696.366087407709 a.u.

C	-0.392676000	-0.236797000	-0.124162000
C	-1.176665000	-2.239760000	-0.792734000
C	0.169988000	-2.292626000	-0.853588000
N	-1.515718000	-0.961892000	-0.345845000
N	0.643972000	-1.051181000	-0.431938000
C	2.055634000	-0.791011000	-0.349457000
C	2.749441000	-1.228632000	0.788494000
C	2.702128000	-0.210233000	-1.447975000

C	4.126242000	-1.027726000	0.818044000
C	4.081159000	-0.040120000	-1.365878000
C	4.787557000	-0.436863000	-0.244313000
H	4.689662000	-1.346994000	1.687684000
H	4.610494000	0.407370000	-2.199508000
C	-2.894859000	-0.639354000	-0.077465000
C	-3.387144000	-0.852003000	1.216326000
C	-3.718705000	-0.245731000	-1.138315000
C	-4.740869000	-0.622016000	1.438593000
C	-5.064593000	-0.021772000	-0.857800000
C	-5.574259000	-0.203922000	0.415254000
H	-5.148126000	-0.774331000	2.431971000
H	-5.724990000	0.298236000	-1.655978000
C	-3.223110000	-0.042844000	-2.555677000
H	-2.172644000	-0.342516000	-2.599518000
C	-2.519515000	-1.314030000	2.367819000
H	-1.485713000	-1.373181000	2.020262000
C	2.081651000	-1.909150000	1.967184000
H	1.018473000	-2.023633000	1.742327000
C	1.982599000	0.217401000	-2.710248000
H	0.908105000	0.094874000	-2.554292000
C	-0.343184000	1.192591000	0.229560000
O	-1.629633000	1.653937000	0.595357000
C	0.655204000	1.684269000	1.216464000
H	-1.781252000	2.354311000	-0.070464000
C	0.507726000	2.142012000	2.490640000
O	1.914494000	1.960701000	0.786775000
C	1.755381000	2.720184000	2.867389000
H	-0.391308000	2.088343000	3.082602000
C	2.572626000	2.574700000	1.798003000
H	2.010272000	3.176928000	3.811514000
H	3.601862000	2.832702000	1.606949000
H	-0.217108000	1.949468000	-0.804252000
O	-0.664645000	2.978366000	-1.542327000
C	-0.059986000	4.167954000	-1.169502000
H	0.262649000	4.178091000	-0.112389000
H	0.841062000	4.398704000	-1.766954000
H	-0.741002000	5.027771000	-1.289185000
C	-2.924656000	-2.707569000	2.848331000
H	-2.267576000	-3.030300000	3.660103000
H	-3.950237000	-2.706133000	3.227318000
H	-2.861656000	-3.446852000	2.046903000
C	-2.556825000	-0.321602000	3.528448000
H	-1.856280000	-0.630583000	4.308498000
H	-2.285570000	0.681984000	3.197919000
H	-3.553306000	-0.275487000	3.975375000

C	-3.306242000	1.427329000	-2.966671000
H	-4.340128000	1.781198000	-2.923142000
H	-2.686474000	2.062520000	-2.331114000
H	-2.958500000	1.543063000	-3.997239000
C	-3.998285000	-0.899627000	-3.557594000
H	-5.040730000	-0.577942000	-3.624804000
H	-3.556350000	-0.797601000	-4.551857000
H	-3.992730000	-1.958027000	-3.290659000
C	2.238972000	1.687669000	-3.035120000
H	1.939785000	2.336847000	-2.211801000
H	3.294982000	1.864451000	-3.254528000
H	1.663182000	1.977233000	-3.917830000
C	2.658969000	-3.301840000	2.223800000
H	2.111764000	-3.783845000	3.037772000
H	2.596101000	-3.948207000	1.346813000
H	3.709168000	-3.240647000	2.520251000
C	2.200026000	-1.069511000	3.238105000
H	1.779510000	-0.072641000	3.102191000
H	1.667915000	-1.559523000	4.057904000
H	3.246768000	-0.963133000	3.535777000
H	-6.626429000	-0.026744000	0.609505000
H	5.861740000	-0.293325000	-0.201641000
C	2.385281000	-0.654332000	-3.900600000
H	3.451232000	-0.544997000	-4.118072000
H	2.185162000	-1.712232000	-3.721518000
H	1.828158000	-0.350308000	-4.790423000
C	-2.182320000	-3.280772000	-1.102071000
H	-2.737419000	-3.053002000	-2.013964000
H	-1.683391000	-4.238410000	-1.244042000
H	-2.907342000	-3.390004000	-0.293496000
C	1.060634000	-3.389413000	-1.292831000
H	1.803985000	-3.640467000	-0.535848000
H	0.468982000	-4.280643000	-1.496690000
H	1.600043000	-3.127466000	-2.205567000

IPr^{Me}-III = Etot =-1696.394068184376 a.u.

C	-0.300112000	-0.475087000	-0.006785000
C	-1.151414000	-2.363931000	-0.955301000
C	0.182854000	-2.412488000	-1.101047000
N	-1.462756000	-1.177263000	-0.273167000
N	0.718805000	-1.241388000	-0.535858000
C	2.122114000	-1.055826000	-0.359444000
C	2.906119000	-0.687749000	-1.465366000
C	2.687542000	-1.291890000	0.901476000
C	4.271894000	-0.514535000	-1.264668000

C	4.057556000	-1.092578000	1.051402000
C	4.844021000	-0.698981000	-0.017071000
H	4.898567000	-0.220821000	-2.099475000
H	4.516568000	-1.265578000	2.018725000
C	-2.779671000	-0.619622000	-0.285191000
C	-3.718744000	-1.041253000	0.667981000
C	-3.112284000	0.304265000	-1.287005000
C	-4.993161000	-0.484010000	0.620540000
C	-4.398108000	0.839742000	-1.285111000
C	-5.331205000	0.456254000	-0.338058000
H	-5.732714000	-0.788014000	1.353168000
H	-4.674342000	1.560378000	-2.047384000
C	-2.146772000	0.715195000	-2.378505000
H	-1.203925000	0.189176000	-2.218889000
C	-3.397059000	-2.053355000	1.747214000
H	-2.394058000	-2.441176000	1.553948000
C	2.330699000	-0.461816000	-2.850726000
H	1.294909000	-0.808928000	-2.847669000
C	1.882990000	-1.802337000	2.078063000
H	0.831771000	-1.826647000	1.786909000
C	-0.251739000	0.711265000	0.708321000
O	-1.365827000	0.982391000	1.510645000
H	-1.150873000	1.200335000	3.359160000
C	0.748721000	1.723800000	0.796694000
C	0.838893000	2.802878000	1.649920000
O	1.762014000	1.789317000	-0.103956000
C	1.982090000	3.547805000	1.239121000
H	0.181185000	3.020640000	2.475097000
C	2.511408000	2.893384000	0.180397000
H	2.364892000	4.454455000	1.683406000
H	3.369298000	3.056498000	-0.450950000
O	-1.078797000	1.316373000	4.322603000
C	-2.058411000	2.249509000	4.725729000
H	-1.961250000	2.392700000	5.803297000
H	-1.924115000	3.222580000	4.237593000
H	-3.075076000	1.894739000	4.517403000
H	-1.875708000	1.709558000	1.129639000
H	-6.328226000	0.883099000	-0.353066000
H	5.909843000	-0.550097000	0.118691000
C	2.296151000	-3.231226000	2.432855000
H	1.676708000	-3.610982000	3.249777000
H	2.183499000	-3.903229000	1.578754000
H	3.339789000	-3.268264000	2.757085000
C	2.001314000	-0.890251000	3.296668000
H	1.697944000	0.130996000	3.057034000
H	1.361123000	-1.257249000	4.103168000

H	3.026990000	-0.863503000	3.674276000
C	-3.385767000	-1.404595000	3.130810000
H	-4.375688000	-1.012961000	3.381957000
H	-3.115462000	-2.143469000	3.890186000
H	-2.670623000	-0.583379000	3.178918000
C	-4.372254000	-3.230416000	1.737374000
H	-4.435773000	-3.704692000	0.755892000
H	-4.052706000	-3.985237000	2.460452000
H	-5.378784000	-2.909376000	2.018346000
C	-2.668019000	0.304018000	-3.755310000
H	-2.858597000	-0.770870000	-3.802483000
H	-3.598546000	0.824001000	-3.998093000
H	-1.933775000	0.554782000	-4.525387000
C	-1.851640000	2.213093000	-2.335597000
H	-1.118997000	2.475653000	-3.103198000
H	-2.755784000	2.798782000	-2.522205000
H	-1.447642000	2.509162000	-1.364501000
C	3.088318000	-1.250293000	-3.919855000
H	2.571753000	-1.164995000	-4.879191000
H	4.100098000	-0.859850000	-4.056091000
H	3.171977000	-2.310417000	-3.670943000
C	2.314461000	1.019339000	-3.230153000
H	1.715029000	1.605061000	-2.534396000
H	3.329143000	1.428062000	-3.233851000
H	1.900917000	1.140435000	-4.235112000
C	1.039558000	-3.487038000	-1.650585000
H	1.779642000	-3.819725000	-0.917078000
H	0.422983000	-4.344055000	-1.919213000
H	1.583451000	-3.171727000	-2.543013000
C	-2.194673000	-3.311048000	-1.407421000
H	-1.740482000	-4.106200000	-1.997340000
H	-2.722076000	-3.771902000	-0.569030000
H	-2.942414000	-2.812727000	-2.031326000

IPr^{Me}-IV Etot = -2039.505275248610 a.u.

C	0.648097000	-1.603017000	-0.316422000
C	-0.482168000	0.333031000	-0.346500000
C	0.803264000	0.612799000	-0.023929000
N	-0.570618000	-1.041131000	-0.516645000
N	1.487942000	-0.590570000	-0.007111000
C	2.869844000	-0.613023000	0.437394000
C	3.892209000	-0.349004000	-0.477810000
C	3.109876000	-0.741205000	1.814127000
C	5.190951000	-0.244683000	0.019743000
C	4.422120000	-0.618349000	2.253577000

C	5.458541000	-0.374851000	1.368038000
H	6.003884000	-0.046591000	-0.669454000
H	4.635401000	-0.712991000	3.311943000
C	-1.883946000	-1.652851000	-0.635572000
C	-2.497342000	-1.770040000	-1.888354000
C	-2.562530000	-1.963644000	0.558631000
C	-3.778290000	-2.319692000	-1.929903000
C	-3.843731000	-2.491403000	0.453883000
C	-4.443013000	-2.688701000	-0.778033000
H	-4.267217000	-2.437870000	-2.889923000
H	-4.384979000	-2.746986000	1.356832000
C	-1.991666000	-1.710802000	1.939638000
H	-0.921368000	-1.519552000	1.841627000
C	-1.896814000	-1.280053000	-3.186995000
H	-0.905330000	-0.878439000	-2.975149000
C	3.677713000	-0.168639000	-1.964281000
H	2.616631000	-0.303940000	-2.180492000
C	2.017712000	-0.990492000	2.831660000
H	1.107688000	-1.263696000	2.295299000
C	1.119154000	-3.061556000	-0.477929000
O	2.247362000	-3.258124000	0.298328000
C	1.465854000	-3.297024000	-1.917894000
C	0.047771000	-4.137763000	0.162015000
H	1.793102000	-3.815922000	1.057690000
C	1.343321000	-2.590082000	-3.069327000
O	2.092612000	-4.472659000	-2.176561000
O	0.529426000	-4.424154000	1.399027000
H	-0.925965000	-3.635761000	0.175682000
C	-0.130938000	-5.370896000	-0.667141000
C	1.908611000	-3.389991000	-4.105843000
H	0.921353000	-1.606596000	-3.180303000
C	2.347870000	-4.518195000	-3.503970000
C	0.205528000	-6.669153000	-0.459606000
O	-0.809545000	-5.256182000	-1.837975000
H	1.985057000	-3.146884000	-5.154161000
H	2.848684000	-5.404608000	-3.859100000
C	-0.282534000	-7.399481000	-1.586584000
H	0.733531000	-7.054726000	0.397306000
C	-0.886536000	-6.493180000	-2.389859000
H	-0.198803000	-8.459928000	-1.769733000
H	-1.401723000	-6.561198000	-3.334638000
H	0.011323000	-5.626683000	2.379532000
O	-0.255461000	-6.326653000	3.035732000
C	0.305992000	-5.995421000	4.280471000
H	1.402850000	-5.948405000	4.244139000
H	-0.059814000	-5.031707000	4.660417000

H	0.028524000	-6.766417000	5.003920000
H	-5.440472000	-3.109926000	-0.835915000
H	6.475441000	-0.282107000	1.733079000
C	2.354466000	-2.151718000	3.764555000
H	1.496558000	-2.369670000	4.405515000
H	2.600773000	-3.056094000	3.205709000
H	3.198398000	-1.910886000	4.415419000
C	1.719300000	0.268570000	3.646857000
H	1.414453000	1.103725000	3.013520000
H	0.913281000	0.071514000	4.358421000
H	2.601855000	0.578032000	4.213556000
C	-2.152919000	-2.906102000	2.875301000
H	-3.204457000	-3.107014000	3.092695000
H	-1.698211000	-3.805297000	2.458009000
H	-1.658796000	-2.689417000	3.825906000
C	-2.631232000	-0.470696000	2.568347000
H	-2.190220000	-0.283842000	3.550704000
H	-2.490050000	0.422642000	1.958515000
H	-3.705743000	-0.622181000	2.703200000
C	-1.762487000	-2.405137000	-4.211032000
H	-1.249765000	-2.038311000	-5.103705000
H	-1.196584000	-3.247777000	-3.813770000
H	-2.747524000	-2.765505000	-4.518384000
C	-2.731762000	-0.142511000	-3.779659000
H	-3.725536000	-0.497178000	-4.064330000
H	-2.856623000	0.683364000	-3.078069000
H	-2.243934000	0.243411000	-4.678271000
C	4.455254000	-1.218247000	-2.756880000
H	4.192780000	-2.230444000	-2.445463000
H	4.233847000	-1.123792000	-3.822604000
H	5.532295000	-1.085735000	-2.625562000
C	4.074129000	1.233631000	-2.426592000
H	3.858601000	1.348524000	-3.491913000
H	3.532672000	2.010552000	-1.884407000
H	5.143939000	1.405628000	-2.282225000
C	1.411378000	1.935675000	0.248718000
H	0.690770000	2.571014000	0.765679000
H	2.304876000	1.851448000	0.865150000
H	1.688002000	2.439186000	-0.680974000
C	-1.606625000	1.280561000	-0.524749000
H	-1.566762000	1.746039000	-1.512874000
H	-2.573304000	0.790483000	-0.420396000
H	-1.540292000	2.077391000	0.217689000

IPr^{Me}-V Etot = -2039.508108591630 a.u.

C	0.612201000	-1.595521000	-0.284854000
C	-0.494191000	0.357291000	-0.346015000
C	0.788443000	0.621201000	-0.002248000
N	-0.597113000	-1.018972000	-0.506429000
N	1.456985000	-0.592188000	0.032902000
C	2.829374000	-0.621454000	0.504716000
C	3.869113000	-0.335127000	-0.384370000
C	3.041350000	-0.751677000	1.886044000
C	5.153660000	-0.207389000	0.144363000
C	4.339644000	-0.604858000	2.357290000
C	5.392297000	-0.335669000	1.498088000
H	5.978754000	0.005556000	-0.525795000
H	4.529491000	-0.701973000	3.420133000
C	-1.917799000	-1.610292000	-0.641169000
C	-2.511155000	-1.727560000	-1.903812000
C	-2.621853000	-1.906426000	0.543107000
C	-3.805429000	-2.242844000	-1.965136000
C	-3.914791000	-2.400363000	0.417564000
C	-4.500498000	-2.583824000	-0.823041000
H	-4.278373000	-2.359213000	-2.933326000
H	-4.475636000	-2.644306000	1.311291000
C	-2.057374000	-1.683932000	1.932265000
H	-0.969689000	-1.639994000	1.855378000
C	-1.866932000	-1.285006000	-3.198166000
H	-0.873660000	-0.897252000	-2.970222000
C	3.691364000	-0.172743000	-1.878445000
H	2.634365000	-0.302308000	-2.117619000
C	1.932899000	-1.039688000	2.874412000
H	1.036719000	-1.301645000	2.310966000
C	1.100633000	-3.072265000	-0.401415000
O	2.161903000	-3.246178000	0.412543000
C	1.459198000	-3.318301000	-1.849494000
C	-0.043175000	-4.080484000	0.131249000
H	1.187886000	-3.916468000	1.491340000
C	1.371062000	-2.611446000	-3.004299000
O	2.089513000	-4.495640000	-2.094178000
O	0.227336000	-4.218370000	1.491763000
H	-1.026723000	-3.635778000	-0.022000000
C	-0.065319000	-5.418524000	-0.524179000
C	1.963502000	-3.411377000	-4.026466000
H	0.955303000	-1.626514000	-3.126242000
C	2.381573000	-4.541592000	-3.413655000
C	0.372945000	-6.644099000	-0.140355000
O	-0.678079000	-5.506028000	-1.731785000
H	2.070347000	-3.167579000	-5.072059000
H	2.887039000	-5.430442000	-3.756036000

C	0.023654000	-7.544243000	-1.193569000
H	0.883081000	-6.875031000	0.780947000
C	-0.607998000	-6.800661000	-2.131205000
H	0.213068000	-8.605741000	-1.241055000
H	-1.056546000	-7.028703000	-3.084721000
H	3.667488000	-3.856332000	0.181013000
O	4.605643000	-4.184487000	0.177683000
C	4.607405000	-5.524754000	0.600004000
H	4.190960000	-5.644585000	1.609305000
H	4.040607000	-6.177116000	-0.077991000
H	5.640470000	-5.881241000	0.620742000
H	-5.508021000	-2.978048000	-0.895382000
H	6.398216000	-0.223992000	1.887523000
C	2.264207000	-2.233845000	3.766801000
H	1.396229000	-2.490983000	4.379282000
H	2.538217000	-3.106358000	3.171502000
H	3.090780000	-2.008726000	4.445365000
C	1.609557000	0.185636000	3.729779000
H	1.305774000	1.040185000	3.121854000
H	0.794148000	-0.044173000	4.420713000
H	2.478961000	0.484172000	4.322161000
C	-2.392941000	-2.818725000	2.896482000
H	-3.457103000	-2.835279000	3.143577000
H	-2.109745000	-3.788479000	2.487552000
H	-1.844812000	-2.672173000	3.830402000
C	-2.546198000	-0.357032000	2.517744000
H	-2.124404000	-0.215772000	3.516141000
H	-2.259049000	0.499497000	1.907504000
H	-3.636079000	-0.359453000	2.608051000
C	-1.727026000	-2.446415000	-4.180185000
H	-1.185787000	-2.120077000	-5.071741000
H	-1.185178000	-3.284057000	-3.740438000
H	-2.710461000	-2.801295000	-4.498887000
C	-2.660704000	-0.151212000	-3.850743000
H	-3.651819000	-0.494889000	-4.157264000
H	-2.791546000	0.697671000	-3.178137000
H	-2.137386000	0.198471000	-4.744160000
C	4.478501000	-1.243048000	-2.633752000
H	4.197474000	-2.247682000	-2.315308000
H	4.288266000	-1.162096000	-3.706682000
H	5.552663000	-1.118582000	-2.472987000
C	4.115291000	1.214429000	-2.360897000
H	3.929828000	1.306480000	-3.434103000
H	3.569123000	2.011125000	-1.853561000
H	5.182707000	1.379194000	-2.192779000
C	-1.604671000	1.313605000	-0.565258000

H	-1.564696000	1.728459000	-1.575650000
H	-2.578783000	0.845434000	-0.431594000
H	-1.522907000	2.145419000	0.135705000
C	1.414377000	1.932157000	0.285140000
H	0.653482000	2.627772000	0.639838000
H	2.191263000	1.850839000	1.044563000
H	1.866021000	2.363956000	-0.610947000

VI... IPr^{Me}-I(H) Etot = -2039.523668970215 a.u. symmetry c1

C	-1.868169000	0.639118000	-0.395752000
C	-2.736912000	1.784150000	-2.169945000
C	-1.409570000	1.626873000	-2.402244000
N	-2.982757000	1.173389000	-0.944182000
N	-0.911159000	0.929432000	-1.305612000
C	0.458234000	0.546060000	-1.153933000
C	1.349362000	1.444790000	-0.553633000
C	0.861199000	-0.715168000	-1.610211000
C	2.668011000	1.032343000	-0.382394000
C	2.191392000	-1.079448000	-1.417305000
C	3.085893000	-0.218817000	-0.804020000
H	3.378044000	1.701328000	0.091048000
H	2.530353000	-2.053538000	-1.752766000
C	-4.271545000	1.103300000	-0.326757000
C	-5.103081000	0.015208000	-0.621521000
C	-4.657351000	2.122260000	0.553241000
C	-6.345613000	-0.039626000	0.004224000
C	-5.910067000	2.021171000	1.152838000
C	-6.747023000	0.951627000	0.883721000
H	-7.007151000	-0.874787000	-0.198121000
H	-6.232677000	2.791808000	1.844310000
C	-3.763214000	3.298756000	0.880826000
H	-2.865916000	3.224021000	0.262710000
C	-4.687970000	-1.094177000	-1.563956000
H	-3.734691000	-0.813530000	-2.017256000
C	0.922279000	2.818657000	-0.081903000
H	-0.091813000	2.999751000	-0.445240000
C	-0.087755000	-1.676769000	-2.293116000
H	-1.052334000	-1.177187000	-2.406035000
H	-1.933663000	-0.282851000	1.124922000
O	-2.105463000	-0.836029000	1.965715000
C	-0.899826000	-1.284602000	2.498254000
C	-0.286750000	-0.280832000	3.483593000
H	-0.160142000	-1.452701000	1.703036000
C	-1.090118000	-2.582044000	3.210199000

O	-0.979710000	0.557336000	4.036428000
C	1.139122000	-0.377045000	3.723113000
C	-1.405077000	-2.910796000	4.489101000
O	-0.990589000	-3.708027000	2.457568000
C	2.131490000	-1.154466000	3.189564000
O	1.670998000	0.460595000	4.654455000
C	-1.510767000	-4.333226000	4.526378000
H	-1.546372000	-2.222454000	5.308384000
C	-1.250727000	-4.759969000	3.268050000
C	3.331894000	-0.771556000	3.830831000
H	2.015063000	-1.908520000	2.426621000
C	2.986265000	0.210984000	4.707026000
H	-1.746570000	-4.952736000	5.377633000
H	-1.209576000	-5.734491000	2.808551000
H	4.321876000	-1.166648000	3.666901000
H	3.555189000	0.802295000	5.407515000
O	-0.425979000	3.031734000	5.282709000
C	-0.205864000	2.896712000	6.670001000
H	-0.521875000	2.142916000	4.901510000
H	-1.039908000	2.392106000	7.172474000
H	0.715196000	2.343176000	6.888711000
H	-0.109990000	3.898428000	7.092774000
H	-7.718235000	0.889915000	1.362638000
H	4.116933000	-0.522617000	-0.658637000
C	-4.469161000	-2.402083000	-0.803890000
H	-5.399912000	-2.744410000	-0.342793000
H	-4.124800000	-3.183033000	-1.487095000
H	-3.723477000	-2.282771000	-0.014929000
C	-5.696073000	-1.290664000	-2.694437000
H	-5.855653000	-0.366091000	-3.254248000
H	-5.332836000	-2.051404000	-3.390155000
H	-6.663755000	-1.625834000	-2.312228000
C	0.402906000	-2.052859000	-3.690296000
H	-0.330263000	-2.696691000	-4.182933000
H	0.552318000	-1.168161000	-4.313638000
H	1.348844000	-2.598982000	-3.645969000
C	-0.314066000	-2.928429000	-1.447254000
H	-0.717303000	-2.676556000	-0.464298000
H	-1.021683000	-3.597067000	-1.944606000
H	0.620736000	-3.476704000	-1.300878000
C	-4.437077000	4.631081000	0.557834000
H	-4.745233000	4.680808000	-0.489185000
H	-5.322478000	4.789682000	1.179020000
H	-3.745414000	5.455680000	0.748470000
C	-3.320552000	3.259090000	2.342961000
H	-2.637309000	4.086023000	2.553882000

H	-4.179380000	3.353971000	3.013338000
H	-2.808345000	2.323962000	2.579131000
C	1.816012000	3.920579000	-0.647216000
H	2.839216000	3.838933000	-0.271818000
H	1.852375000	3.886786000	-1.738692000
H	1.431894000	4.899580000	-0.349769000
C	0.881442000	2.887839000	1.443717000
H	0.186413000	2.153120000	1.854401000
H	1.870547000	2.697623000	1.870112000
H	0.558351000	3.880110000	1.769608000
C	-3.790116000	2.445298000	-2.975216000
H	-3.361293000	2.845363000	-3.893729000
H	-4.585139000	1.746778000	-3.248695000
H	-4.253170000	3.271749000	-2.429747000
C	-0.566235000	2.061340000	-3.540247000
H	0.206896000	2.766480000	-3.223618000
H	-0.063149000	1.213427000	-4.012152000
H	-1.181068000	2.552158000	-4.294383000
